# Accurate prediction of RNA secondary structure including pseudoknots through solving minimum-cost flow with learned potentials

**DOI:** 10.1038/s42003-024-05952-w

**Published:** 2024-03-09

**Authors:** Tiansu Gong, Fusong Ju, Dongbo Bu

**Affiliations:** 1 Key Lab of Intelligent Information Processing, Institute of Computing Technology, Chinese Academy of Sciences, 100190 Beijing, China; 2 University of Chinese Academy of Sciences, 100190 Beijing, China; 3 Central China Artificial Intelligence Research Institute, Henan Academy of Sciences, Zhengzhou, 450046 Henan China

**Keywords:** Computational biology and bioinformatics, Computational models

## Abstract

Pseudoknots are key structure motifs of RNA and pseudoknotted RNAs play important roles in a variety of biological processes. Here, we present KnotFold, an accurate approach to the prediction of RNA secondary structure including pseudoknots. The key elements of KnotFold include a learned potential function and a minimum-cost flow algorithm to find the secondary structure with the lowest potential. KnotFold learns the potential from the RNAs with known structures using an attention-based neural network, thus avoiding the inaccuracy of hand-crafted energy functions. The specially designed minimum-cost flow algorithm used by KnotFold considers all possible combinations of base pairs and selects from them the optimal combination. The algorithm breaks the restriction of nested base pairs required by the widely used dynamic programming algorithms, thus enabling the identification of pseudoknots. Using 1,009 pseudoknotted RNAs as representatives, we demonstrate the successful application of KnotFold in predicting RNA secondary structures including pseudoknots with accuracy higher than the state-of-the-art approaches. We anticipate that KnotFold, with its superior accuracy, will greatly facilitate the understanding of RNA structures and functionalities.

## Abstract (web summary)

Presenting KnotFold, an application for accurately predicting RNA secondary structures, including pseudoknots.

## Introduction

Ribonucleic acid (RNA) are polymer molecules with essential roles in a large variety of biological processes^1,2^, including transcription, translation^3^, catalysis^4^, gene expression regulation^5^, protein synthesis^6^, and degradation^7^. Most biologically active RNAs, say mRNA, tRNA, and non-coding RNAs (ncRNAs), usually fold into specific structures due to the existence of self-complementary parts formed by base pairing. These structures, together with RNA primary sequences, largely determine the biological functions of RNAs^8^; thus, a deep understanding of RNA structures is of great significance.

RNA structures can be experimentally determined using X-ray crystallography^9^, nuclear magnetic resonance^10^, or cryo-electron microscopy^11^. Besides, RNA secondary structures, which typically form through pairing certain bases with hydrogen bonds and are stable and accessible in cells^12,13^, can be experimentally investigated using enzymatic and chemical probing methods^14,15^. These experimental determination technologies have achieved great progress; however, the high experimental cost usually required by these technologies^16^ precludes their applications – over 24 million ncRNAs have been sequenced and collected in the RNAcentral database^17^ but only a tiny fraction of them have their structures experimentally determined^18^. Compared with these experimental determination technologies, computational prediction of RNA structures purely from RNA sequences is substantially efficient and has become a promising method for understanding RNA structures.

Thermodynamic models are commonly employed in RNA secondary structure prediction, as they quantify the stability of an RNA structure by calculating its folding free energy change and then select the structure with the lowest free energy or the maximum expected accuracy as the most probable one in the entire structure ensemble^19–21^. Turner’s nearest-neighbor model^21^, a representative of thermodynamic prediction approaches, decomposes an RNA secondary structure into a collection of nearest-neighbor loops. It characterizes these loops by using multiple free energy parameters and then sums up the parameters to obtain the free energy of the entire secondary structure^22,23^. The free energy parameters are typically determined using experimental techniques, such as optical melting^24^, or determined statistically through machine learning analysis of known RNA structures^25–27^. The optimal base pairs for the secondary structure with the lowest free energy or the maximum expected accuracy can then be calculated using the recursive dynamic programming technique^28^. The representative prediction approaches using this strategy include mfold^19^, RNAfold^29^, and RNAstructure^30^.

**Fig. 1:**
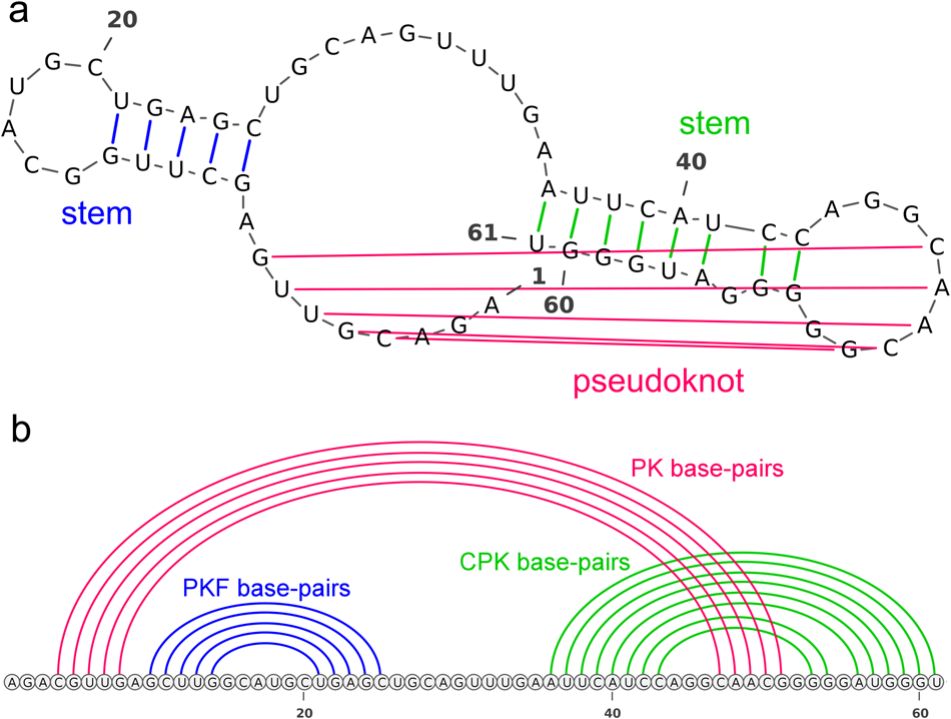
An example of RNA secondary structure including pseudoknots (CP000097.1_937913-937973). **a** The RNA secondary structure includes a pseudoknot formed by five base pairs: 4C-51G, 5G-50C, 6U-49A, 7U-48A, and 8G-47C. **b** Base pairs are divided into three categories for better evaluation of structures including pseudoknots: (*i*) pseudoknotted (PK) base pairs (in magenta), (*ii*) pseudoknot-free (PKF) base pairs (in blue), and (*iii*) crossing-pseudoknot (CPK) base pairs (in green).

RNA structures often contain a unique type of structure motif known as pseudoknots (Fig. 1), which are bipartite helical structures formed by pairing a single-stranded region inside a stem-loop structure with a complementary stretch outside^31^. Pseudoknots can serve as standalone elements or parts of complex RNA structures for stabilization^32,33^, replication, RNA processing, inactivation of toxins, and gene expression control^34–36^. Thus, understanding pseudoknots is important.

Despite the importance of pseudoknots, accurately predicting RNA secondary structure including pseudoknots is challenging, partly due to the various composition of loops and helices and the lack of sequence-specific features^37^. Calculating the lowest free energy pseudoknotted structure under the nearest neighbor model has been proved to be NP-hard^38^. To address this problem, conventional prediction approaches make compromises by limiting pseudoknot types or even focusing on pseudoknot-free structures only. However, even when imposing reasonable limitations on pseudoknot types, the conventional dynamic programming algorithms still need *O*(*L*^4^) ~ *O*(*L*^6^) time for an RNA with *L* bases, thereby precluding their applications for medium or large RNAs^39–42^. Other approaches, such as ILM^43^, HotKnots^44^, FlexStem^45^, ProbKnot^46^, BiokoP^47^, and IPknot^48,49^, circumvent this computation difficulty using heuristic strategies. Although these approaches are relatively fast, they often cannot guarantee the quality of the predicted secondary structures. Recently, deep learning has been applied to predict base pairing probabilities with promising results. The popular deep learning-based approaches include SPOT-RNA^50^, E2Efold^51^, and UFold^52^. However, constructing the secondary structure from the predicted base pairing probability remains a challenge.

In this study, we report an accurate and fast approach (called KnotFold) to the prediction of RNA secondary structure including pseudoknots. KnotFold comprises two key elements:

(1) *A structural potential learned using attention-based neural network:* KnotFold learns a structural potential from known RNA structures, i.e., it predicts the base pairing probabilities for any two bases using an attention-based neural network and then transforms the predicted probabilities into a potential function. This strategy reduces the inaccuracies of hand-crafted free energies as it is learned from a large number of RNAs with known structures. Unlike the nearest-neighbor model calculating the contribution of a base pair to free energy according to its neighboring base pairs, the self-attention mechanism enables KnotFold to capture the relationship between bases, including long-distance interactions and non-nested base pairs, thus making it more suitable for identifying pseudoknots.

(2) *A specially designed minimum-cost flow algorithm to find the secondary structure with the lowest potential:* We realize the structure with the lowest potential by solving a minimum-cost network flow problem. Briefly, the network uses nodes to represent bases and uses edges to represent base pairs with appropriate capacity and cost for each edge according to the calculated potential values. This way, KnotFold can consider all possible combinations of base pairs rather than posing constraints on pseudoknot types.

We evaluated KnotFold on multiple benchmark sets, including PKTest (1009 RNAs), TS0 (1305 RNAs), and RfamNew (472 RNAs). KnotFold exhibits performance improvements in predicting pseudoknots, particularly for pseudoknotted base pairs (see Section for the classification of base pairs and Fig. 1 b for an example). We anticipate that the enhanced accuracy of KnotFold will contribute to comprehending RNA structures and functionalities.

## Results

In this section, we first demonstrate the concept of KnotFold using RNA CP000097.1_937913-937973 (retrieved from Rfam^53^) as a representative, and then exhibit the performance of KnotFold on three benchmarks (including PKTest, TS0, and RfamNew), and compare it with existing approaches as well. We further construct a confidence index that measures the reliability of the predicted secondary structures. We also analyze the role of the key elements of KnotFold through examining the running process of KnotFold on a representative RNA.

### Overview of the KnotFold approach

KnotFold predicts the secondary structure of a target RNA through three main steps, i.e., predicting the base pairing probabilities for any two bases of the given RNA, constructing a potential function using the acquired base pairing probabilities, and realizing the optimal secondary structure with the lowest potential using a specially designed minimum-cost flow algorithm. We describe these steps in detail as follows.

#### Learning the base pairing probability

For an RNA sequence *x* with *L* bases, we describe one of its possible secondary structures as an *L* × *L* matrix *S* = {*S*_*ij*_∣*S*_*ij*_ ∈ {0, 1}, 1 ≤ *i*, *j* ≤ *L*}, where *S*_*ij*_ = 1 if the *i*-th base and the *j*-th base form a base pair in the secondary structure and *S*_*ij*_ = 0 otherwise. To find the most likely secondary structure for the target RNA sequence, we first apply an attention-based deep neural network to predict the base pairing probability *P*(*bp*_*i*,*j*_∣*x*) for any two bases *i* and *j*. In particular, the neural network uses transformer encoder layers^54^ to encode bases and then calculates the outer product of the encoding of any two bases, which is further projected as an *L* × *L* matrix, representing the pairing probabilities *P*(*bp*_*i*,*j*_∣*x*). The use of self-attention mechanism gains our approach an advantage that, when predicting the pairing probabilities between two bases, the entire sequence including long-range interactions, rather than these two bases alone, is taken into consideration.

#### Constructing structural potential considering all pairs of bases

To measure the likelihood of a secondary structure *S* for an RNA with its sequence *x*, we construct an RNA-specific structural potential from the predicted base pairing probabilities *P*(*bp*_*i*,*j*_∣*x*) as follows:

1 $$\,{{\mbox{E}}}(S,x)= 	-\mathop{\sum}\limits_{\begin{array}{c}i < j\\ {S}_{i,j}=1\end{array}}{{\mbox{log}}}\frac{P(b{p}_{i,j}| x)}{P(b{p}_{i,j}| {{\mbox{length}}})}\\ 	-\mathop{\sum}\limits_{\begin{array}{c}i < j\\ {S}_{i,j}=0\end{array}}{{\mbox{log}}}\frac{1-P(b{p}_{i,j}| x)}{1-P(b{p}_{i,j}| {{\mbox{length}}}\,)}+\lambda \mathop{\sum}\limits_{i < j}{S}_{ij}.$$

Here, the first and second term account for the contributions by each base pair, with *P*(*bp*_*i*,*j*_∣length) representing the reference base pairing probabilities employed to rectify the over-representation of the prior. The third term introduces a penalty on unsuitable structures exhibiting either an excess or a deficiency of base pairs, with *λ* representing the weight of this term. We provide the details of the reference base pairing probabilities in Methods and the discussion on *λ* in Supplementary Text.

#### Calculating the optimal secondary structure

To find the optimal secondary structure *S* that minimizes the potential E(*S*, *x*), KnotFold solves a modified minimum-cost flow problem^55–57^, in which the optimal flow corresponds to the optimal secondary structure. Briefly speaking, we first construct a bipartite graph, in which both parts consist of *L* nodes, and each node corresponds to a base of the given RNA. We draw an edge from each node in the left part to each node in the right part. We further add an extra node (called *source node*, denoted as *s*) and connect it to each node in the left part. Similarly, we also add an extra node (called *sink node*, denoted as *t*) and connect it by each node in the right part. The key point of KnotFold is that, by setting an appropriate capacity and cost for each edge according to the calculated potential values, the minimum-cost flow for this network-flow problem exactly corresponds to the secondary structure with the lowest potential. The algorithm to solve the minimum-cost flow, together with the setting of capacities and costs for edges, are described in more detail in Methods.

**Fig. 2:**
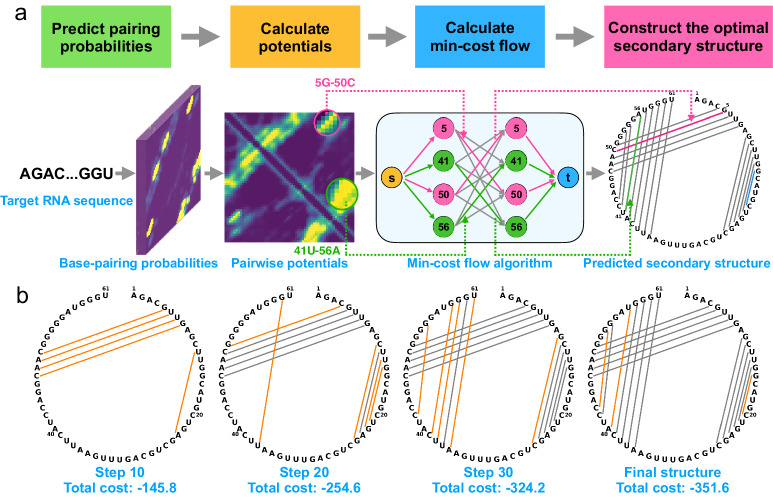
Overview of the KnotFold approach to predicting RNA secondary structure including pseudoknots. **a** The main procedures of KnotFold illustrated using CP000097.1_937913-937973 as an example: KnotFold first predicts the base pairing probabilities for any two bases of the target RNA, then constructs a potential function based on the acquired base pairing probability, and finally realizes the optimal secondary structure with the lowest potential using the specially designed minimum-cost flow algorithm. Here, the flow network shows four bases, i.e., 5G, 41U, 50C, 56A, and 12 edges among these bases as representatives, and KnotFold selects the corresponding base pairs 5G-50C (in magenta) and 41U-56A (in green) as part of the predicted secondary structure. The final prediction consists of a total of 18 base pairs but only one false-positive base pair 15G-20C (in blue). **b** The iteration steps of solving the optimal flow. The proposed algorithm begins with a zero flow with no edges and iteratively adds new edges to the current flow, or sometimes removes existing edges. We use KnotFold to construct the secondary structures corresponding to the intermediate flows. The cost decreases as iteration proceeds and finally reaches −351.6 after 36 steps. During this process, some base pairs are newly added (shown as orange lines here) while some are removed, which is described in more detail in Supplementary Fig. 5.

Using RNA CP000097.1_937913-937973 (containing 61 bases, see Fig. 2a) as a representative of pseudoknotted RNA, we demonstrate the basic idea and main concepts of KnotFold as follows:

First, using a deep neural network, KnotFold predicts the base pairing probability and calculates the potential accordingly. As shown in Fig. 2, the potential exhibit three strips with low values. These strips, which are perpendicular to the main diagonal, provide strong signals of three possible base pair stackings formed by the base pairing between the regions [4, 8] and [47, 51], [10, 15] and [20, 25], and [36, 43] and [53, 61], respectively.

Next, KnotFold constructs a flow network with associated cost and capacity on edges. Each edge’s capacity is set as 1, thus posing a restriction that a base can pair with at most one base, and the edge’s costs are set according to the learned potential function. For example, the edges 5G-50C and 41U-56A are assigned with a negative cost of −8.84 and −0.47, respectively. In contrast, the edges 5G-41U and 41U-50C have a positive cost of 11.93 and 9.35, respectively.

Then, KnotFold calculates the minimum-cost flow using the specially designed algorithm described in Methods. Here, the cost of a flow is defined as the sum of costs over all edges traveled by the flow. To solve the minimum-cost flow, the algorithm begins with a zero flow and continuously improves the current flow by adding, removing, or replacing some edges, in the hope of decreasing the total cost of the flow step by step. In the case of RNA CP000097.1_937913-937973, after a total of 36 steps of improvement, the algorithm eventually acquired the minimum-cost flow with a total cost of −351.6, among which 5G-50C and 56A-41U are saturated, whereas 5G-41U and 50C-41U are empty edges (Fig. 2b).

Finally, we obtained a predicted secondary structure using the edges traveled by the optimal flow, i.e., selecting the saturated edges with the flow value of 1. In this case, KnotFold reported 18 base pairs, including 5G-50C and 41U-56A, and successfully identified the pseudoknot (Fig. 2).

### Performance evaluation and comparison with existing approaches over pseudoknotted RNAs

We evaluated KnotFold and nine existing approaches in terms of the accuracy of the predicted secondary structure and base-pair probabilities. We further investigated how pseudoknot types and RNA length affect these predictions.

#### Secondary structure prediction accuracy for pseudoknotted RNAs

To evaluate the performance of KnotFold on pseudoknot identification, we constructed a dataset (PKTest) by randomly selecting 1009 pseudoknotted RNAs from bpRNA^58^ and Rfam 14.5^53^, with a nucleotide length limit of 500. We performed filtering such that the sequence identity between PKTest and the training set is below 80%, thus avoiding the overlap between training and test sets (see Methods for details of dataset construction).

**Fig. 3:**
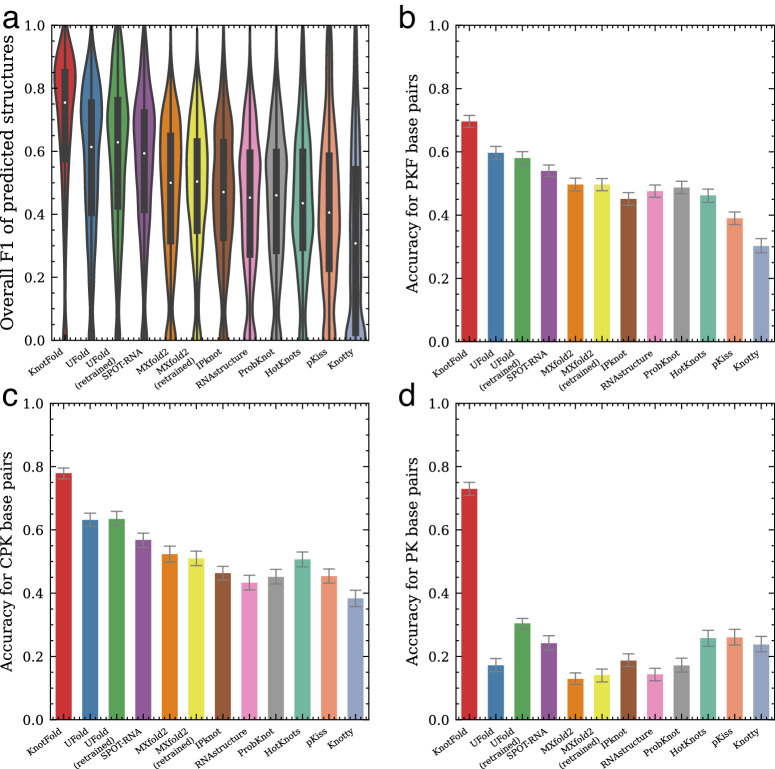
Performance of KnotFold and nine existing approaches to RNA secondary structure prediction over PKTest data set. **a** The overall F1 scores of predicted structures by various approaches evaluated on the PKTest dataset. **b** The prediction accuracy of RNA secondary structure prediction approaches for pseudoknot-free base pairs. **c** The prediction accuracy of RNA secondary structure prediction approaches for crossing-pseudoknot base pairs. **d** The prediction accuracy of RNA secondary structure prediction approaches for pseudoknotted base pairs. Error bars in **b**–**d** represent standard errors.

We compared KnotFold with nine widely-used RNA secondary structure prediction methods, including RNAstructure^30^ ProbKnot^46^, HotKnots^44^, pKiss^42^, Knotty^59^, IPknot^48,49^, SPOT-RNA^50^, MXfold2^60^ and UFold^52^. These approaches, except MXfold2 and RNAstructure, support pseudoknot prediction. Among these approaches, ProbKnot, pKiss, Knotty, HotKnots, and IPknot use dynamic programming or heuristic method to predict structures with the minimum free energy or the maximum expected accuracy, whereas UFold and SPOT-RNA fold structures using rule-based strategies from base pairing probabilities predicted by deep learning techniques. In the case of MXfold2 and UFold, we initially assessed their released versions. However, the performance of deep learning models is affected by training data size, thus we mitigated potential biases and ensure fair comparison by retraining MXfold2 and UFold with the same dataset utilized by KnotFold. We provide the details of these experiments in Supplementary Text. Fig. 3a and Supplementary Table 1 suggest that KnotFold exhibits superior performance compared to all other approaches on the PKTest dataset, as demonstrated by its average F1 score. Specifically, KnotFold achieves a high F1 score of 0.758, followed by UFold (retrained version) with an F1 score of 0.602. SPOT-RNA achieves an F1 score of 0.579, and MXfold2 (retrained version) with an F1 score of 0.498.

#### Base-pair-level evaluation on pseudoknotted RNAs

The structural configuration of pseudoknots poses a challenge to bio-computational detection due to its “overlapping" nature^38^. Unlike other base pairs, pseudoknot base pairs are not well nested and occur overlap one another in sequence positions. This feature makes it difficult for standard methods, such as dynamic programming algorithms, to detect non-nested base pairs. To address this challenge, we classify base pairs in the target structures into three categories based on their structural motifs, inspired by IPknot^48^: (*i*) pseudoknot-free (PKF) base pairs, which do not cross with any other base pair, (*ii*) pseudoknotted (PK) base pairs, which are the minimum set of base pairs that, if removed, the remaining secondary structure would be pseudoknot-free, and (*iii*) crossing-pseudoknot (CPK) base pairs, which cross with some pseudoknotted base pairs (see Fig. 1b for an example). For the three categories of base pairs, pseudoknot-free base pairs are nested base pairs, whereas crossing-pseudoknot and pseudoknotted base pairs are non-nested. Detecting these non-nested base pairs, particularly pseudoknotted base pairs, is crucial for accurate structure prediction approaches.

To better evaluate the performance of RNA secondary structure prediction methods on structures containing pseudoknots, we conduct a base-pair-level analysis on the PKTest dataset. As summarized in Fig. 3 and Supplementary Table 2, KnotFold achieves the accuracy of 0.697, 0.783, and 0.734 for pseudoknot-free, crossing-pseudoknot, and pseudoknotted base pairs, respectively. Interestingly, the majority of current methods demonstrate satisfactory performance when predicting pseudoknot-free and crossing-pseudoknot base pairs, but less so for pseudoknotted base pairs. Specifically, the prediction accuracy for most techniques surpasses 0.4 for pseudoknot-free and crossing-pseudoknot base pairs but falls short of 0.25 for pseudoknotted base pairs. KnotFold, however, offers a more consistent performance across all base pair types, boasting a 37% improvement over the next-best approach, pKiss, with an accuracy of 0.260 for the pseudoknotted base pair category. As a result, KnotFold outperforms other approaches across the three base pair categories, with its superiority being particularly evident for crossing-pseudoknot and pseudoknotted base pairs.

We further observe that approaches designed for pseudoknot prediction do better in pseudoknotted base pair identification. For example, although pKiss and Knotty exhibit modest accuracy levels for pseudoknot-free base pairs (0.454 and 0.382, respectively) and crossing-pseudoknot base pairs (0.390 and 0.301, respectively), they achieve relatively high accuracy for pseudoknotted base pairs prediction (0.260 and 0.236, respectively). In contrast, MXfold2 and RNAstructure yield poor performance for pseudoknotted base pair prediction, with respective accuracies of 0.137 and 0.144. This performance difference may lie in the construction strategy of these approaches. For example, the dynamic programming algorithm used by MXfold2 and RNAstructure, which takes a recursive scoring system to identify paired stems and consequently, fails in detecting non-nested base pairs.

#### The prediction accuracy on RNAs with various pseudoknot types

Pseudoknots can be classified according to its complexity, and complicated pseudoknots are commonly regarded more difficult to predict. Here, we adopted the complexity category proposed by M Kucharík, et al., which classifies pseudoknots into four types, including H-type (involving a loop and a single-stranded region outside the loop), K-type (involving interactions between loops), as well as more intricate L-type and M-type pseudoknots^61^. According to this category, 720 out of the 1009 pseudoknotted RNAs in PKTest are classified as H-type, 218 as K-type, and 71 as L- or M-type.

We summarized the performance of KnotFold and other nine approaches in Supplementary Table 1. As shown in this table, KnotFold achieves F1 scores of 0.713, 0.782, and 0.783 for H-type, K-type, and L- or M-type pseudoknots, respectively. It is noteworthy that many established prediction methods, including SPOT-RNA, IPknot, pKiss, and Knotty, work well on the H-type and K-type pseudoknots but relatively worse on the L- or M-type pseudoknots.

Furthermore, we exhibited two solid cases in Supplementary Fig. 1, where KnotFold succeeds in identifying both K-type and L-type pseudoknots. These instances highlight KnotFold’s unique strength in addressing complex RNA structures that are traditionally hard to predict.

#### The prediction accuracy for RNAs with various length

**Fig. 4:**
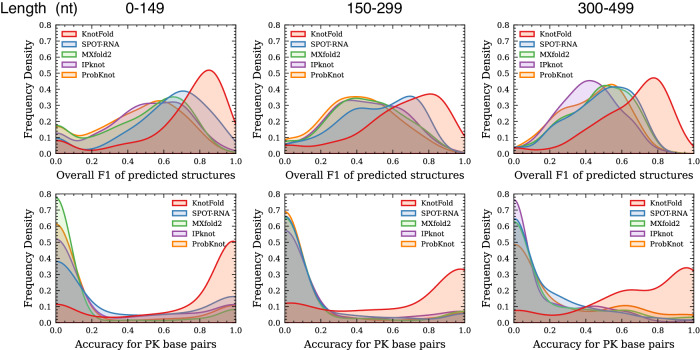
Performance of RNA secondary structure prediction approaches over PKTest. The frequency density of the overall F1 and the accuracy of pseudoknotted base pairs of five approaches including KnotFold, SPOT-RNA, MXfold2, IPknot, and ProbKnot are evaluated over three subgroups of PKTest, which are divided by RNA lengths.

To assess the accuracy of RNA secondary structure prediction for various RNA lengths, we stratify the RNA sequences in PKTest into three groups based on length: 535 RNAs ranging from 0 to 149 nt, 229 RNAs ranging from 150 to 299 nt, and 255 RNAs ranging from 300 to 499 nt. For each RNA length group, we depicted the frequency density of the overall F1 score and prediction accuracy for pseudoknotted base pairs in Fig. 4. To facilitate clearer visual comparisons, we presented the results of the most accurate five approaches, including KnotFold, SPOT-RNA, MXfold2, IPknot, and ProbKnot. KnotFold demonstrated F1 scores of 0.708 (with improvements of 0.102), 0.642 (0.133 improvements), and 0.646 (0.167 improvements) for RNAs with lengths in the ranges of 0–149, 150–299, and 300–499, respectively. Concurrently, KnotFold achieved an accuracy of 0.763 (0.428 improvements), 0.673 (0.527 improvements), and 0.701 (0.553 improvements) for pseudoknotted base pairs in each group. The results show that KnotFold’s advantage is more pronounced for longer RNAs, indicating its ability of identifying long-distance and non-nested base pairs.

**Fig. 5:**
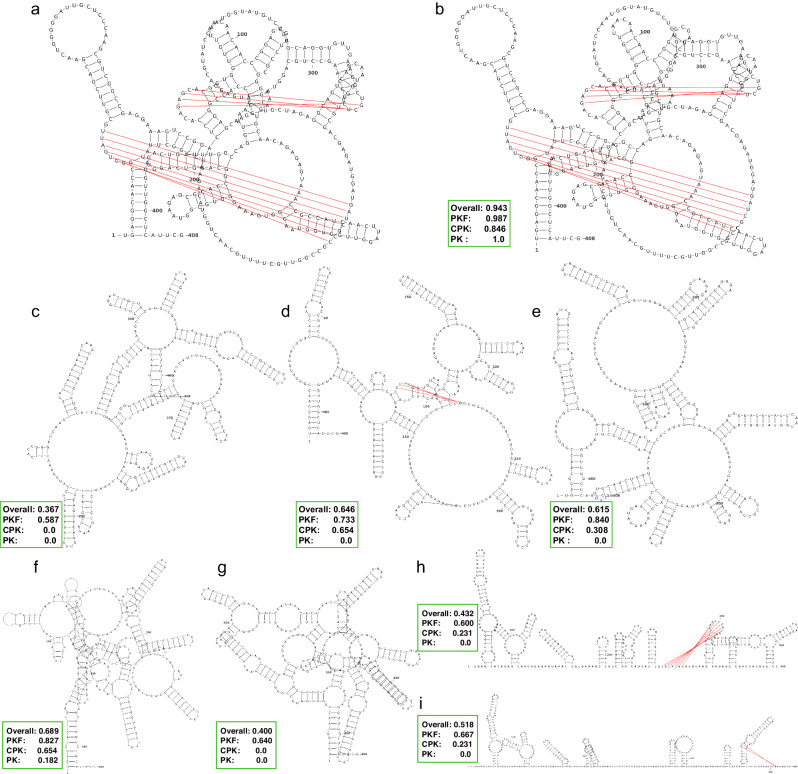
Predicting secondary structure of AP009044.1_2333762-2333355 using KnotFold and nine existing approaches. **a** The ground-truth secondary structure of the target RNA, with pseudoknotted base pairs shown in red. The predicted structures by KnotFold (**b**), ProbKnot (**c**), SPOT-RNA (**d**), MXfold2 (**e**), UFold (**f**), HotKnots (**g**), IPknot (**h**), and Knotty (**i**) have accuracies of 0.943, 0.367, 0.646, 0.615, 0.689, 0.400, 0.432, and 0.518, respectively. KnotFold identifies all pseudoknotted base pairs (in red) and 84.6% of crossing-pseudoknot base pairs.

Figure 5 provides a concrete example: AP009044.1_2333762-2333355 is a 408-nucleotide-long RNA containing five large bulges together with two pseudoknots, one connecting regions [12, 18] and [349, 355], while the other connects regions [79, 82] and [289, 292]. UFold, ProbKnot and MXfold2 report secondary structures with five, four and three bulges, respectively; however, neither of them correctly identified the pseudoknots. IPknot, SPOT-RNA, and Knotty only successfully identified one pseudoknot each. Additionally, RNAstructure, pKiss, and UFold reported structures with F1 scores of 0.367, 0.383, and 0.689. In contrast, KnotFold successfully identified both the five large bulges and the two pseudoknots, achieving a high prediction accuracy of 0.943. A similar observation can be made for another pseudoknotted RNA, TRW-314253_1-314 (see Supplementary Fig. 2 for further details).

Overall, KnotFold shows superiority in improving RNA secondary structure prediction across different RNA lengths and base pair categories, particularly for challenging pseudoknotted structures.

### Assessing the robustness of KnotFold

To investigate the robustness of KnotFold, we performed two types of cross validation as conducted by MXfold2^60^, including sequence-wise cross-validation, in which individual RNA sequences are partitioned into test datasets and training datasets, and family-wise cross-validation, in which test RNAs belong to different families as RNAs in training datasets.

#### Sequence-wise cross-validation

We evaluated KnotFold and the existing approaches on TS0, a subset extracted from bpRNA-1m^58^ by SPOT-RNA^50^. Specifically, SPOT-RNA first extracts 13,419 non-redundant RNAs with length less than 500 bases from by running CD-HIT-EST^62^, and randomly splits them into three datasets, including training set TR0 (10,814 RNAs), validation set VL0 (1300 RNAs), and test set (1305 RNAs, containing 129 pseudoknotted RNAs). For fair comparison, we trained KnotFold on SPOT-RNA’s training set TR0 and then evalutated its performance alongside existing approaches on the test set TS0. The existing approaches were executed with their default parameter settings.

**Fig. 6:**
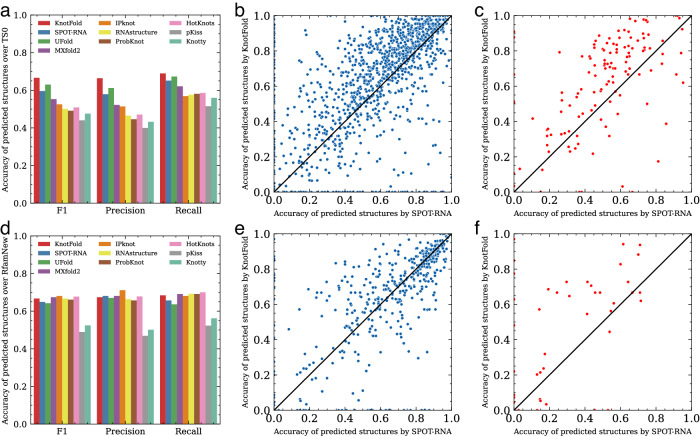
Sequence-wise and family-wise evaluation of KnotFold and nine other RNA secondary structure prediction approaches. **a** The overall F1 scores of predicted structures by various approaches over TS0 (1305 RNAs). **b** Head-to-head comparison of built structures of SPOT-RNA and KnotFold over TS0. **c** Head-to-head comparison of built structures of SPOT-RNA and KnotFold over pseudoknot targets in TS0. **d** The overall F1 scores of predicted structures by various approaches over RfamNew (472 RNAs). **e** Head-to-head comparison of built structures of SPOT-RNA and KnotFold over RfamNew. **f** Head-to-head comparison of built structures of SPOT-RNA and KnotFold over pseudoknot targets in RfamNew.

As shown in Fig. 6 and Supplementary Table 3, KnotFold achieves a high F1 score of 0.666, followed by UFold with an F1 score of 0.630, and SPOT-RNA with an F1 score of 0.596. Detailed comparison of the accuracy of each target structure constructed by SPOT-RNA and KnotFold (Fig. 6b, c) indicates that KnotFold outperforms SPOT-RNA for most target RNAs (67.1%), and this ratio is even higher for the pseudoknotted targets (76.0%).

#### Family-wise cross-validation

To test whether these approaches can predict RNAs from new families, we collected 1435 sequences from 168 newly added RNA families after the release of Rfam 14.5. After removing redundant sequences at 80% sequence-identity cut-off using CD-HIT-EST^62^, we acquired 472 RNAs (containing 51 pseudoknotted RNAs) and used them to build a test set RfamNew (see Methods for more details of RfamNew). This data process guarantees that the test set RfamNew has no overlap with the training set at the family level. As sequence-wise cross-validation, the existing approaches were executed with their default parameter settings.

As shown in Fig. 6d–f and Supplementary Table 4, KnotFold, together with nine existing approaches, exhibited perfect prediction accuracy. KnotFold achieved an F1 score of 0.667, slightly lower than IPknot (0.681) and MXfold2 (0.674). Head-to-head analysis suggested that the advantage of KnotFold is much more clear in the pseudoknotted RNAs. We also observed that IPknot, MXfold2, and RNAstructure achieved relatively high accuracy on RfamNew than that on TS0 (Fig. 6d). The underlying reason might be that these approaches emphasize the common characteristics shared by various RNA families.

### Constructing confidence index for secondary structure prediction

For a prediction approach, an important issue is whether we can judge the quality of its prediction results in advance. When the target structure of RNA is already known, we can easily evaluate a predicted structure through comparing it with the target structure; however, this issue becomes challenging when the target structure is not available. Here we present an index that measures the confidence of the predicted secondary structure for an RNA.

**Fig. 7:**
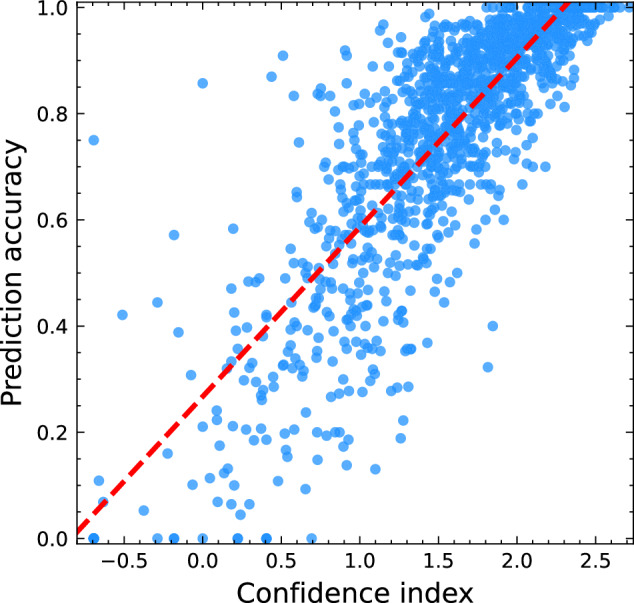
Correlation between the prediction accuracy and the estimated confidence index. We use the value of average cost on saturated edges as the confidence index (logarithmically transformed). For the 1131 RNAs in the validation dataset, the Pearson correlation coefficient between the prediction accuracy (F1 score) and confidence index reaches 0.836.

We constructed a confidence index according to the high correlation between the average cost of saturated edges in the optimal flow reported by KnotFold and the accuracy of secondary structure prediction. Here, an edge is called saturated if it is traveled by the minimum-cost flow, which exactly means the two bases corresponding to this edge form a base pair in the predicted secondary structure. We observed that, on the 1131 RNAs in the validation set, the Pearson’s correlation coefficient between the log average cost over saturated edges and the prediction accuracy (F1 score) achieves 0.836 (Fig. 7). This strong correlation enabled us to use the log average cost over saturated edges as a confidence index for the prediction. For example, when setting the confidence index cut-off as 1.35, most of KnotFold’s predictions are highly accurate: for 717 out of 755 RNAs, the prediction accuracy exceeds 0.60 (see Supplementary Fig. 7 for further details). The proposed confidence index provides an effective way to assess the reliability of RNA structure predictions.

### Assessing the role of minimum-cost flow algorithm in KnotFold

To assess the role of the key elements of KnotFold, we developed a variant of KnotFold as control (called KnotFold-DP), which uses the same potential function as KnotFold but employs dynamic programming technique instead of minimum-cost flow to find the optimal secondary structure (see Supplementary Text for further details of KnotFold-DP).

For the potential function we constructed (Eq. (1)), the minimum-cost flow algorithm constructs an arbitrary secondary structure with the lowest potential. However, when restricting RNAs to nested base pairs only, i.e. non-pseudoknot RNAs, a dynamic programming algorithm could be applied to construct the lowest potential structure.

We observed that KnotFold-DP can accurately predict secondary structure for non-pseudoknot RNAs. In particular, sequence-wise and family-wise evaluation indicate that KnotFold-DP gains 0.005 improvements for the non-pseudoknot RNAs in TS0 and 0.008 improvements for the non-pseudoknot RNAs in RfamNew.

**Fig. 8:**
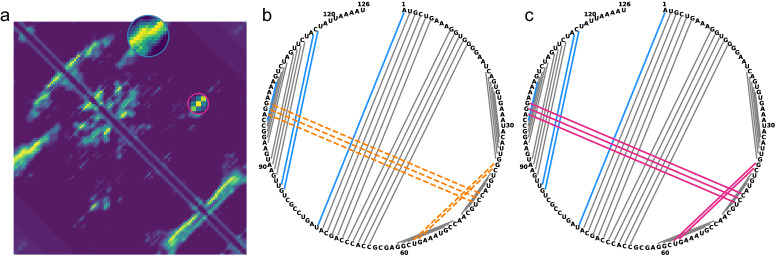
The difference between KnotFold and its variant KnotFold-DP illustrated using AACY020619257.1_605-730 as an example. Both KnotFold and KnotFold-DP use the same potential as their input, and they differ only in the algorithms to find the secondary structure: KnotFold uses a specially designed minimum-cost flow algorithm while KnotFold-DP uses the dynamic programming algorithm. **a** The calculated potential for the target RNA. Here, circles highlight two regions of base pairs that are crossing. **b** The predicted secondary structure by KnotFold-DP. The orange dash lines represent the missing base pairs, while the blue lines represent the false-positive base pairs. **c** The predicted secondary structure by KnotFold. The base pairs missed by KnotFold-DP are successfully predicted (shown in magenta).

However, KnotFold-DP showed poor performance on pseudoknotted RNAs. Specifically, on PKTest dataset, KnotFold-DP achieved an accuracy of 0.716 for pseudoknot-free base pairs, 0.698 for crossing-pseudoknot base pairs, and only 0.176 for pseudoknotted base pairs, which is 55.8% less than that by KnotFold. A failure case of KnotFold-DP is shown in Fig. 8: for RNA AACY020619257.1_605-730, all five pseudoknotted base pairs (shown in magenta) were completely missed by KnotFold-DP; in contrast, KnotFold can successfully identify these base pairs. The failures can be attributed to the fact that the dynamic programming algorithm is recursive, making it suitable for nested base pairs. However, pseudoknotted base pairs defy this recursion assumption, resulting in the identification of only a subset of base pairs by KnotFold-DP.

Moreover, the minimum-cost flow algorithm could further enhance the predictions of other methods based on the prediction of base pair probabilities. For example, Supplementary Tables 7 and 8 demonstrates that the application of the minimum-cost flow algorithm can enhance the predictions of SPOT-RNA.

These findings highlight the role of minimum-cost flow algorithm and the broader utility of the minimum-cost flow algorithm in improving the prediction of pseudoknotted RNA structures.

## Discussion

The results presented in this study emphasize the unique features of KnotFold: KnotFold employs a deep neural network to learn a structural potential considering all base pair interactions, making it suitable for identifying long-distance interactions and non-nested base pairs, particularly pseudoknots. Additionally, KnotFold utilizes a specially designed minimum-cost algorithm to determine the secondary structure with the lowest potential. Through popular benchmark datasets, we demonstrate KnotFold’s accuracy and superiority over the existing approaches. In addition, we propose a confidence index for evaluating the prediction accuracy of RNA secondary structures even when the ground-truth is unknown. This confidence index facilitates robust assessments of the reliability of KnotFold’s predictions.

KnotFold’s ideas can be readily extended without significant modifications to solve other complex structural motifs. For instance, by changing the edge capacity from 1 to 2, KnotFold can predict secondary structures including base triples^63^, an important RNA structural motif involving three bases interacting edge-to-edge through hydrogen bonding^64,65^. The benchmark tests and case studies shown in Supplementary Tables 12, 13, and Supplementary Fig. 3, 4 demonstrate that, the enhanced KnotFold successfully identified base triples and predicted RNA secondary structures including base triples with high accuracy. This advantage will greatly facilitate the understanding of RNA functions.

KnotFold constructs secondary structures using a specially designed minimum-cost flow algorithm from base pairing probabilities learned by neural networks. To investigate the advantages of our proposed algorithm over the rule-based strategies, we examined the middle results of the proposed algorithm. As shown in Supplementary Fig. 5, the proposed algorithm sometimes removes certain base pairs that have been tentatively selected using “backward flows", a unique feature of the network flow algorithm, thereby obtaining the optimal secondary structure. Furthermore, the structure ensembles constructed by these tentative flows yield a series of sub-optimal secondary structures, which may provide a deep insight into the predicted secondary structure.

The parameter *λ* in Eq. (1) is introduced to control the number of base pairs expected to appear in the predicted RNA secondary structure, achieving a trade-off between precision and recall of predictions. As demonstrated in Supplementary Fig. 6a, when setting *λ* as 4.2, the precision and recall over the validation set achieve a balance. As the setting of *λ* is strongly associated with the number of base pairs, we can modify the objective function to treat *λ* as a function of the number of base pairs. Specifically, the optimal *λ* displays a strong linear correlation with RNA length (Pearson correlation coefficient: 0.988, as illustrated in Supplementary Fig. 6b). This finding allows us to appropriately set *λ* prior to running KnotFold according to RNA length.

The generalization ability for new RNA families structure prediction is a common challenge for deep learning based methods. To assess this issue, we conducted a series of experiments. Family-wise cross-validation experiments on RfamNew revealed that the performance of deep learning models, including KnotFold, fell short compared to existing physics-based models (e.g., IPknot). Similar results were observed on the TORNADO dataset (see Supplementary Table 9 for details). The underlying reasons may be the susceptibility to overfitting of deep learning models, and the low data coverage and density over diverse structures, as discussed in earlier studies^66,67^. To address this challenge, potential solutions include: 1) Actively exploring the integration of inductive bias terms, such as statistical energy terms, into the KnotFold model; 2) Considering the implementation of more ensemble strategies to introduce greater diversity to the models. This approach has proven effective in mitigating overfitting (see Supplementary Tables 5 and 6 for further details); and 3) Viable efforts towards the incorporation of relevant data. Beyond experimental determinations of secondary structures, high-throughput experimental data such as SHAPE^68^ and PARIS^69^ yield nucleotide-level activity profiles, presenting an opportunity for integration^70^. As part of future work, we aim to enhance the model’s robustness across RNA families.

To assess performance changes with lower sequence identity, we evaluated KnotFold on the bpRNA dataset. KnotFold exhibited diminished performance (0.761 to 0.512) when trained and tested on datasets with lower sequence identity cut-offs (0.80 to 0.40), as shown in Supplementary Table 10. This highlights the common challenge for computational models under stricter sequence identity cut-offs, leading to decreased performance. In ongoing efforts, we are actively exploring strategies to improve the model’s performance under these conditions.

We anticipate that KnotFold, with its superiority in accuracy and efficiency, will greatly facilitate our understanding of RNAs with complicated structures and their biological functions.

## Methods

### The KnotFold framework

The main steps of KnotFold are described in Algorithm 3, involving three main steps: 1) predicting base pairing probabilities for any two bases in the target RNA using an attention-based neural network, 2) calculating structural potential from these predicted probabilities, and 3) constructing the optimal secondary structure with the lowest potential using a specially designed minimum-cost flow algorithm.

The workflow of KnotFold [1] Target RNA sequence *x* The secondary structure *S*^*opt*^ with the lowest potential Predict base pairing probabilities *P*(*bp*_*i*,*j*_∣*x*) by the prior neural network; Predict reference probabilities *P*(*bp*_*i*,*j*_∣length) by the reference neural network;

Construct a flow network *G* with edge capacities and costs calculated according to *P*(*bp*∣*x*) and *P*(*bp*∣length); Calculate the lowest potential structure *S*^*opt*^ through solving the minimum-cost flow;

### Predicting base pairing probabilities using an attention-based neural network

#### Network architecture

We propose an attention-based prior neural network to predict base pairing probabilities *P*(*bp*_*i*,*j*_∣*x*) (Supplementary Fig. 8). The prior neural network takes an RNA sequence of length *L* as its sole input, and outputs an *L* × *L* matrix of base pairing probabilities. The network comprises two main steps, i.e., encoding bases using transformer encoder layers^54^, and predicting the base pairing probabilities from the base encoding thus acquired. These two steps are described in more detail below.

Initially, each base type (A, C, G, U, or N) is converted into a learnable embedding of *D* dimensions using an embedding layer. This RNA sequence embedding, denoted as $Z\in {{\mathbb{R}}}^{L\times D}$, is then processed through a stack of transformer encoder layers. Transformer is an attention-based architecture that can efficiently capture long-range dependencies within sequences^54^. Each transformer encoder layer includes a multi-head self-attention module and a position-wise feed-forward network. By stacking several such layers, the prior model effectively captures the context and dependencies of bases within the given RNA sequence. As highlighted by Vaswani et al.^54^, the transformer architecture possesses a permutation-invariant nature; thus, we supplement the sequence embedding with relative positional encodings^71^.

Subsequently, the prior model predicts base pairing probabilities based on the base encoding thus acquired. We calculate the outer product of the *L* × *D* representation, obtaining a *L* × *L* feature map denoted as $g(i,j)\in {{\mathbb{R}}}^{D\times D},1\le i,j\le L$. Specifically, for the embedding features of base *i* (${t}_{i}\in {{\mathbb{R}}}^{D}$) and base *j* (${t}_{j}\in {{\mathbb{R}}}^{D}$), their outer product is calculated as:

2 $$g(i,j)={t}_{i}\otimes {t}_{j}\,.$$

Here, “ ⊗ ” denotes the outer product operation. Finally, a regression layer is applied to each *g*(*i*, *j*), transforming the *D* × *D* representation into the probability that the *i*-th and *j*-th base form a base pair.

To correct the over-estimation of the prior probabilities, we also calculate the reference probability *P*(*bp*_*i*,*j*_∣length) using a reference neural network, which has the same architecture as the prior network but uses the length of RNA instead of the entire sequence.

#### Loss function

To ensure that the predicted base pairing probabilities closely resemble the ground-truth secondary structure, we calculate the loss function using binary cross-entropy over all possible base pairs as follows:

3 $$\,{{\mbox{Loss}}}\,=-\mathop{\sum}\limits_{i < j}[{P}^{* }(b{p}_{i,j})\,\,{{\mbox{log}}}\,P(b{p}_{i,j}| x)+(1-{P}^{* }(b{p}_{i,j}))\,\,{{\mbox{log}}}\,(1-P(b{p}_{i,j}| x))].$$

Here, P^*^(*bp*_*i*,*j*_) = 1 if bases *i* and *j* form a base pair in the ground-truth structure, and P^*^(*bp*_*i*,*j*_) = 0 otherwise. This loss function measures the dissimilarity between the predicted base pairing probabilities and the ground-truth secondary structure.

#### Hyperparameter setting and training setup

For the balance of performance and model size, we set the embedding dimension *D* as 256, and use 8 transformer encoder layers with 8 attention heads in the study, resulting in a total of 6.5 million parameters. To fit the memory limitation, we crop the RNA sequence to a 512 base fragment randomly if its length exceeds 512 bases during training.

We train KnotFold with a batch size of 4 RNAs for 300,000 steps on GPU (Tesla V100 PCIe, 16GB memory). We use AdamW^72^ with a learning rate of 0.001, *β*_1_ = 0.9, *β*_2_ = 0.999, L2 weight decay of 0.01, learning rate warm-up over the first 30,000 steps, and linear decay of the learning rate. To reduce the influence of the bias of training sets, we employ a model ensemble strategy for KnotFold by calculating the average of base pairing probabilities from five randomly initialized prior models.

### Calculating the potentials from base pairing probabilities

We develop an RNA-specific structural potential, denoted as E(*S*, *x*), to assess the likelihood of a secondary structure *S* for the target RNA sequence *x* (Eq. (1)). Briefly speaking, the potential function considers the accumulated contribution by all possible pairs of bases. In Eq. (1), the first and the second term calculate the negative log-likelihood of the relative base pair probabilities for all pairs of bases in the given secondary structure, whereas the third term penalizes the inappropriate number of base pairs in the secondary structure.

In fact, this potential function is designed to conform to the loss function, as Eq. (3) can be reformulated as

$\,{{\mbox{Loss}}}=-\mathop{\sum}\nolimits_{i < j,{S}_{i,j}^{truth}=1}{{\mbox{log}}}P(b{p}_{i,j}| x)-\mathop{\sum}\nolimits_{i < j,{S}_{i,j}^{truth}=0}{{\mbox{log}}}\,(1-P(b{p}_{i,j}| x))$, with *S*^*truth*^ refers to the ground-truth secondary structure.

This way, the secondary structure *S* with the lowest potential E(*S*, *x*) essentially describes the the most likely base pairs.

### Constructing the flow network *G*

To realize the secondary structure with the lowest potential, we calculate the minimum-cost flow of a network, in which the edge cost and capacity are appropriately set such that the optimal flow corresponds to the secondary structure with the lowest potential. Briefly speaking, the minimum-cost flow problem is an optimization and decision problem to find the cheapest possible way of sending a certain amount of flow through a flow network^73–75^. In this problem, each edge in the network has an associated cost, and the goal is to find the flow with the minimal cost.

The construction of the flow network *G* is described in Algorithm 3. In particular, we initialize a network consisting of a bipartite, in which each part of the bipartite consists of *L* nodes that represent the *L* bases of the target RNA. Each node in the left part is connected to each node in the right part with an edge, which essentially represents a possible base pair. For the edge (*i*, *j*) connecting the *i*-th base and the *j*-th base, we set its capacity as 1 and its cost as *H*(*i*, *j*), the value of which is described as follows.

4 $$H(i,j)=-[\,{{\mbox{log}}}\frac{P(b{p}_{i,j}| x)}{P(b{p}_{i,j}| {{\mbox{length}}})}-{{\mbox{log}}}\frac{1-P(b{p}_{i,j}| x)}{1-P(b{p}_{i,j}| {{\mbox{length}}}\,)}]+\lambda .$$

By setting the edge’s capacity as 1, the flow value of each edge in *G* is either 0 or 1, i.e., an edge should be either saturated (flow value is 1) or empty (flow value is 0)^74^. This property ensures that, in this flow network, the optimal flow essentially describes the secondary structure with the lowest potential, and each saturated edge exactly corresponds to a base pair in the predicted structure. A detailed derivation process is provided in Supplementary Text.

Construct a flow network *G* [1] RNA length *L*, edge costs *H*(*i*, *j*) Flow network *G* Add *L* nodes to the left part *V*_*left*_ and *L* nodes to the right part *V*_*right*_ Add two extra nodes: source node *s* and sink node *t* each node *i* ∈ *V*_*left*_ Add an edge (*s*, *i*) with a cost of 0 and a capacity of 1 each node *j* ∈ *V*_*right*_ Add an edge (*j*, *t*) with cost 0 and capacity 1 each node *i* ∈ *V*_*left*_ each node *j* ∈ *V*_*right*_ Add an edge (*i*, *j*) with a cost of *H*(*i*, *j*) and a capacity of 1

### Solving the optimal flow using a specially designed minimum-cost algorithm

We solve the minimum-cost flow in the constructed flow network *G* using a modified minimum-cost flow algorithm (Algorithm 3). Specifically, we start from a 0-flow, i.e., all edges are initialized with a flow value of 0. Next, we iteratively execute the following two steps:

(i) Constructing a residual graph *G*_*f*_ according to the current flow *f*. For each edge (*i*, *j*) in the flow network, we add two edges into the residual graph *G*_*f*_, including a forward edge (*i*, *j*) with capacity 1 − *f*(*i*, *j*) and cost *H*(*i*, *j*), and a backward edge (*j*, *i*) with capacity *f*(*i*, *j*) and cost − *H*(*i*, *j*).
(ii) Finding the shortest path from the source *s* to the sink *t*, denoted as *s* ⇝ *t*, in the residual graph *G*_*f*_, followed by pushing along this path to augment the current flow *f*. Here, the shortest *s* ⇝ *t* path refers to the path with the minimum accumulated cost of the edges traveled by this path.

Finally, we extract the saturated edges from the optimal flow, i.e., the edges with a flow value of 1, and report a secondary structure with base pairs corresponding to these saturated edges as the predicted secondary structure.

Construct the secondary structure with the lowest potential using the specially designed minimum-cost flow algorithm [1] Initialized flow network *G* The optimal secondary structure *S*^*opt*^ Set the initial flow *f* as 0 the residual graph *G*_*f*_ contains an *s* ⇝ *t* path with negative cost Select a shortest *s* ⇝ *t* path *P* Augment the current flow *f* along the path *P* Update the residual graph *G*_*f*_ Construct *S*^*opt*^ from saturated edges

This proposed algorithm is derived from the classical successive shortest path algorithm, proposed by Ford et al.^55–57^. Briefly, the successive shortest path algorithm iteratively augments a flow along the cheapest path from the source *s* to the sink *t* that has non-zero capacity until no more augmenting paths can be found.

Unlike the classical algorithm, we use a modified stopping criterion by early stopping the algorithm if no *s* ⇝ *t* path with negative cost exists in the residual network *G*_*f*_. As illustrated by Ford et al.^55^, the proposed algorithm maintains a feasible flow *f* with the lowest cost among all possible flows with the same size as *f*. This property ensures our proposed algorithm reaches the optimal flow corresponding to the secondary structure with the lowest potential with the change of stopping criterion.

Theoretically speaking, the proposed algorithm runs efficiently, with a time complexity of *O*(*L*^3^) for an RNA with *L* bases. As highlighted in previous works^74,75^, the flow augmentation (line 2 of Algorithm 3) is performed for no more than *L* times, and each augmentation process costs *O*(*L*^2^) time, which is dominated by finding the shortest path. Despite the theoretical time complexity, the algorithm runs extremely fast in practice due to the unit capacity of edges and the bipartite graph. For example, for an RNA with less than 1000 bases, the proposed algorithm can construct a secondary structure from base pairing probabilities within 20 s (Intel CPU 2.6GHz). We provide the practical running time details for different RNA lengths of our proposed algorithm in Supplementary Fig. 9.

### Datasets and data processing

#### Datasets

In this study, we assessed the performance of various prediction approaches using RNA extracted from several databases. Specifically, we evaluated the following datasets:

(i) bpRNA-1m: This comprehensive dataset contains 102,318 RNA secondary structures from various sources, including Rfam (version 12.2). We used bpRNA-1m for training, testing, and validation purposes^58^.
(ii) Rfam: This database contains 3940 RNA families (version 14.5). We used RNAs released after the version 12.2 to extend our training, testing, and validation datasets^53^.
(iii) PKTest: We created this dataset by randomly extracting 1009 pseudoknot RNA sequences with no longer than 500 bases from bpRNA-1m and Rfam 14.5. There are 535, 229 and 255 RNAs with lengths between 0 and 149, between 150 and 299, and between 300 and 499, respectively (see Supplementary Table 11 for further details). We used PKTest to evaluate the approaches’ ability to identify general pseudoknots.
(iv) TS0: This dataset is a subset of bpRNA-1m extracted by SPOT-RNA^50^. It contains 1305 RNAs, including 129 pseudoknot RNAs.
(v) RfamNew: This dataset collects 472 non-redundant RNAs from the 168 RNA families newly added to Rfam 14.9 after the release of version 14.5 (see Supplementary Table 11 for further details). We used this dataset for family-wise validation.

#### Data processing

We used non-redundant RNAs collected in bpRNA-1m^58^ and Rfam 14.5^53^ to prepare the training, validation, and test sets for KnotFold. We first removed redundant sequences using CD-HIT-EST^62^ with a cut-off threshold of 80% sequence-identity. After that, we randomly selected 1009 RNAs with pseudoknots in their secondary structures whose lengths are less than 500 from the non-redundant RNAs as a pseudoknot test set PKTest. The remaining RNAs are split into a training set and a validation set, comprising 23,819 and 1131 RNAs, respectively.

To evaluate RNA secondary prediction approaches on new RNA families, we constructed an RNA family test set RfamNew by collecting RNAs that belong to different families with the 23,819 training RNAs. We initially collected 1435 RNA sequences from the 168 families that are newly added to Rfam 14.9 after the release of version 14.5. Next, we removed sequences with sequence similarity of more than 80% using CD-HIT-EST. After removing sequence similarity, 472 sequences remained, which are used to form RfamNew.

The training and test datasets are available in Supplementary Data 1.

### Evaluation criteria

We evaluated the accuracy of the prediction accuracy through precision, recall and F1 score of the base pairs, defined as

5 $$\begin{array}{rlr}\,{{\mbox{Precision}}}\,&=\frac{\,{{\mbox{TP}}}}{{{\mbox{TP}}}+{{\mbox{FP}}}\,},&\\ \,{{\mbox{Recall}}}\,&=\frac{\,{{\mbox{TP}}}}{{{\mbox{TP}}}+{{\mbox{FN}}}\,},\\ \,{{\mbox{F1}}}\,&=\frac{2\times \,{{\mbox{Precision}}}\times {{\mbox{Recall}}}}{{{\mbox{Precision}}}+{{\mbox{Recall}}}\,},\end{array}$$

where TP is the number of correctly predicted base pairs (true positives), FP is the number of incorrectly predicted base pairs (false positives), and FN is the number of base pairs in the reference structure that were not predicted (false negatives).

We calculated the average precision, recall, and F1 score to evaluate the overall performance on a dataset and the average of recall to evaluate the performance on different types of base pairs on PKTest.

### Reporting summary

Further information on research design is available in the Nature Portfolio Reporting Summary linked to this article.

### Supplementary information

Supplementary Information

Description of Supplementary Materials

Supplementary Data 1

Reporting Summary

## Data Availability

Our training and test data splits are available with this paper. The following versions of public datasets were used in this study: bpRNA-1m 1.0 (https://bprna.cgrb.oregonstate.edu/); and RFAM 14.9 (https://rfam.org/).

## Code availability

All source codes and models of KnotFold are publicly available through https://github.com/gongtiansu/KnotFold.
