# Supplementary material for: Accurate prediction of RNA secondary structure including pseudoknots through solving minimum-cost flow with learned potentials
Source: Commun Biol. 2024 Mar 9;7:297. doi: 10.1038/s42003-024-05952-w (PMC10924946; doi:10.1038/s42003-024-05952-w)
Supplement: Supplementary file 1 — Supplementary Information [file 42003_2024_5952_MOESM1_ESM.pdf]

Accurate prediction of RNA secondary structure including pseudoknots through solving minimum-cost flow with learned potentials

Tiansu Gong[1], Fusong Ju[1] and Dongbo Bu[1,2*]

[1]Key Lab of Intelligent Information Processing, Institute of Computing Technology, Chinese Academy of Sciences, Beijing 100190, China.
[2]University of Chinese Academy of Sciences, Beijing 100190, China.
[3]Central China Artificial Intelligence Research Institute, Henan Academy of Sciences„ Zhengzhou 450046, Henan, China.

*Corresponding author(s). E-mail(s): dbu@ict.ac.cn;
Contributing authors: gongtiansu19z@ict.ac.cn;
jufusong@ict.ac.cn;

Contents

1 Supplementary results and discussions **4**
- 1.1 Experimental settings for secondary structure prediction methods 4
- 1.2 Extending KnotFold to identify base triples 4
- 1.3 Analysis on ensemble strategy 5
- 1.4 Assessing the prediction accuracy of base pair probability . . . 6
- 1.5 Removing the tentatively-selected base pairs during the execution of KnotFold 6
- 1.6 Optimal setting of the parameter λ in potential function 7
- 1.7 Investigate KnotFold's robustness using TestSetA and TestSetB 8
- 1.8 Evaluate the performance of secondary structure prediction at lower sequence identity 8
- 1.9 Enhancing the predictions of SPOT-RNA using the minimum-cost flow algorithm 9

2 Supplementary methods **10**
- 2.1 Reformulating the potential function as the total cost of a flow 10
- 2.2 The construction of the variant KnotFold-DP 11
- 2.3 The construction of the variant KnotFold-LP 11

3 Supplementary figures and tables **12**

Supplementary Figure 1. The predicted secondary structures by KnotFold on two RNAs with K- and L-type pseudoknots
Supplementary Figure 2. Predicted secondary structures by KnotFold, MXfold2, ProbKnot, SPOT-RNA, UFold and IPknot (target RNA: `TRW-314253_1-314`)
Supplementary Figure 3. Predicted RNA secondary structures including base triples using the enhanced KnotFold (RNA: PDB entry `5L4O`)
Supplementary Figure 4. Predicted secondary structures including base triples using the enhanced KnotFold (RNA: PDB entry `7LYJ`).
Supplementary Figure 5. Removing tentatively-selected base pairs for improving prediction (target RNA: `BA000035.2_1474355-1474472`)
Supplementary Figure 6. Optimal setting of parameter λ in the structural potential
Supplementary Figure 7. The prediction accuracy at different confidence index cut-offs
Supplementary Figure 8. The network architecture used by KnotFold for predicting base pairing probability
Supplementary Figure 9. The running time of the minimum-cost flow algorithm for RNAs with different length in the validation set

Supplementary Table 1. The accuracy of secondary structure prediction approaches for different complexity of pseudoknot structures

on PKTest
Supplementary Table 2. The predicted accuracy of secondary structure prediction approaches for pseudoknot-free base pairs, crossing-pseudoknot base pairs, and pseudoknotted base pairs on PKTest
Supplementary Table 3. The predicted accuracy of the secondary structure prediction approaches evaluated on TS0
Supplementary Table 4. The predicted accuracy of the secondary structure prediction approaches evaluated on RfamNew
Supplementary Table 5. The prediction accuracy of the five models within the ensemble of identical network architectures on the validation set, PKTest, and RfamNew
Supplementary Table 6. The model architecture and the prediction accuracy of the five models within the ensemble of different network architectures on the validation set, PKTest, and RfamNew
Supplementary Table 7. Accuracy of the predicted base pair probabilities generated by KnotFold's prior model, SPOT-RNA, and UFold on PKTest
Supplementary Table 8. Accuracy of the predicted secondary structure by KnotFold, SPOT-RNA and their variants on PKTest.
Supplementary Table 9. The predicted accuracy of KnotFold, TORNADO, ContextFold, CONTRAfold, MXfold, and MXfold2.
Supplementary Table 10. The predicted accuracy of KnotFold at different sequence identity thresholds using CD-HIT and MMseqs2
Supplementary Table 11. The number of pseudoknot base pairs in PKTest and RfamNew
Supplementary Table 12. The number of base triples in TR1, VL1, TS1 and TS2
Supplementary Table 13. Predicted accuracy of KnotFold and SPOT-RNA on TS1 and TS2

1 Supplementary results and discussions

1.1 Experimental settings for secondary structure prediction methods

To mitigate biases introduced by training data and ensure a fair comparison, we trained models of KnotFold, MXfold2, and UFold on the constructed training set with Intel CPU version Xeon E5-2690 v4 (2.60G Hz, 64GB memory) and GPU version Tesla V100 PCIe (16GB memory). We employed the default hyperparameters to train MXfold2 for 10 epochs and UFold for 100 epochs. We evaluated the performance of KnotFold and eight other approaches over PKTest, including SPOT-RNA, MXfold2 (the released and the retrained versions), UFold (the released and the retrained versions), RNAstructure, ProbKnot, IPknot, Knotty, and pKiss. For pKiss, we utilized the enforced mode instead of the mfe mode for generating structures containing pseudoknots. The other approaches were run with default experimental settings.

We also evaluated the performance of BiokoP [1], which utilizes a bi-objective integer programming algorithm to optimize both energy and probabilistic criteria in RNA secondary structure prediction. Regrettably, BiokoP could only predict the secondary structure of a small subset of the RNAs (86 out of 1305 on TS0, for example), accounting for less than 10% of the dataset, and the average accuracy of BiokoP over reported structures reached a middle level (0.535) among these RNA structure prediction approaches. As a result, we excluded the performance of BiokoP from our analysis.

1.2 Extending KnotFold to identify base triples

Besides base pairs, an RNA might also form base triples [2], which involve three bases interacting edge-to-edge by hydrogen bonding. Supplementary Figure 3 shows the secondary structure of the RNA with PDB entry `5L4O`, which contains three base triples, `25C-10G-45G` (in magenta), `13C-22G-46A` (in green), and `37A-29G-41C` (in blue). Previous studies have reported the importance of base triples in RNA structures and functions [3, 4].

The conventional dynamic programming algorithms, however, do not allow for base triples when constructing secondary structures. In contrast, KnotFold is capable to predict secondary structures including base triples with a slight modification without any change of its essence. In particular, we enhance KnotFold by changing the edge capacity from 1 to 2, which allows a base to interact with 2 other bases, thus forming base triples.

As shown in Supplementary Figure 3, the original KnotFold missed the base pair `25C-10G` (shown as magenta dashed line), and thus failed to identify the base triple `25C-10G-45G`. Similarly, the absence of base pairs `22G-46A` and `29G-41C` leads to the failure in identifying the other two base triples `13C-22G-46A` and `37A-29G-41C`. In contrast, the enhanced KnotFold successfully identified all three base triples, and thus correctly predicted the secondary

structure for this RNA. A similar result is illustrated for another RNA `7LYJ` in Supplementary Figure 4.

To benchmark KnotFold's performance for base triple prediction, we utilize datasets collected from PDB by SPOT-RNA. The datasets include a training set TR1, a validation set VL1, and two test sets TS1 and TS2, which contain 104, 27, 53, and 29 RNAs with base triples, respectively (see Supplementary Table 12 for details). The training set TR1 has limited number of sequences, and it alone is insufficient for training. To overcome this difficulty, we employed the transfer learning strategy as performed by SPOT-RNA. In particular, we first trained KnotFold using bpRNA and Rfam 14.5 dataset, and then fine-tuned the model using TR1.

We assessed KnotFold and SPOT-RNA on the test sets TS1 and TS2. As shown in Supplementary Table 13, KnotFold achieves an accuracy of 0.739 on TS1, higher than SPOT-RNA by 0.025. The predicted accuracy of KnotFold for base triples reaches 0.239, higher than SPOT-RNA by 0.047. The advantage of KnotFold over SPOT-RNA is much clearer on the TS2 dataset. These results clearly illustrate KnotFold's capacity in predicting RNA base triples.

1.3 Analysis on ensemble strategy

In the paper, an ensemble of five randomly initialized models is used to increase robustness against overfitting in the calculation of base-pairing probabilities. Each model has the same network structure, but differs only in the initial values of the parameters. It is crucial to understand the accuracy of each model and how the ensemble strategy affects the predictions.

To investigate the ensemble strategy, we carried out two experiments, one using five models with the same network architecture but different initial parameters, and the other using five models with different network architectures, i.e., the number of Transformer layers, and the embedding dimension. The network architectures as well as the experimental results are summarized in Supplementary Table 5 and 6. From these tables, we achieved the following observations:

- In the case that five models share an identical network architecture but different initial parameters, the individual models exhibit similar performance. For example, on the validation set containing 1,131 RNAs, the F1 scores of the five models are 0.715, 0.711, 0.712, 0.717, and 0.712, respectively. The ensemble of these five models exhibits an F1 score of 0.724, slightly higher than the best-performing individual model by 0.007.
- In the case that five models have different network architectures, their performance showcases relatively large variation. For example, on the validation set, the five models achieve an F1 score of 0.721, 0.714, 0.717, 0.720, and 0.709, respectively. The ensemble of these five models exhibit an additional improvement in accuracy, achieving an F1 score of 0.732, even higher than the ensemble with the same network structure by 0.008.

We further repeated these experiments on a sequence-level test set PKTest (containing 1,009 pseudoknotted RNAs) and a family-level test set RfamNew (containing 472 RNAs extracted from the 168 RNA families newly added to Rfam 14.9) and observed similar phenomena (see Supplementary Table 5 and 6 for further details).

Together, these results suggest that, compared with the models sharing identical network architecture, the models using different architectures can yield better ensemble models. These observations are consistent with the view point by T G Dietterich [5], i.e., different network architectures introduce more varieties to the individual models, which will facilitate the ensemble strategy.

1.4 Assessing the prediction accuracy of base pair probability

We evaluate the accuracy of the base pair probability directly generated by the neural networks. For the sake of fair comparison, we used the same evaluation metric as SPOT-RNA, i.e., treating the task as a binary classification problem (forming base pairs or not) on the upper triangle probability matrix. As illustrated by Supplementary Table 7, the F1 score of the base pair probability generated by KnotFold's prior model reaches 0.729 on the PKTest dataset, higher than SPOT-RNA by 0.161. Similar results can be found when comparing KnotFold with UFold as well as its retrained version. The results highlight the advantage of KnotFold in predicting base pairing probabilities.

We also investigated the power of self-attention network used by KnotFold in predicting pseudoknotted base pairs on PKTest. As shown in Supplementary Table 7, KnotFold achieves an F1 score of 0.697, 0.771, and 0.687 for pseudoknot-free, crossing-pseudoknot, and pseudoknotted base pairs, respectively. In contrast, SPOT-RNAachieved the F1 score of 0.552, 0.596, and 0.226, respectively. These results highlight the advantages of its self-attention network architecture used by KnotFold in predicting pseudoknotted base pairs.

Furthermore, we evaluated whether KnotFold's self-attention networks have advantage in the identification of long-distance base pairs. Here, similarly to Ref. [6], we defined "long-distance base pairs" as these pairs at least 101 bases apart in sequence, and we adopted the average accuracy for long-distance base pairs as our evaluation criteria.

As shown in Supplementary Table 7, on PKTest, KnotFold achieves the accuracy of long-range interactions of 0.535, significantly higher than SPOT-RNA (0.285). We observed similar phenomena from the comparison on the validation set. These results clearly demonstrate KnotFold's capacity in predicting long-distance base pairs.

1.5 Removing the tentatively-selected base pairs during the execution of KnotFold

To identify the base pairs with the lowest potential, the minimum-cost flow algorithm used by KnotFold sometimes removes certain base pairs that

have tentatively been selected. KnotFold accomplishes this objective using "backward flows", a unique feature of the network flow algorithm.

Supplementary Figure 5 shows one iteration step of KnotFold when predicting secondary structure for `BA000035.2_1474355-1474472`. After selecting 22 base pairs, the minimum-cost flow algorithm reports a tentative secondary structure with a cost of -379.829. At this stage, the minimum-cost flow algorithm found an improvement path that contains two backward edges `15G-40C` and `23G-36C` (shown as blue lines). After removing the base pairs corresponding to these two edges, KnotFold obtained an improved secondary structure with a lower potential of -379.831. This removing operation is key to the success of KnotFold in acquiring the optimal secondary structure.

1.6 Optimal setting of the parameter λ in potential function

As shown in Equation 1, the parameter λ is set to control the number of base pairs expected to appear in the predicted RNA secondary structure. We set the optimal λ as 4.2 such that the average number of base pairs expected to appear in the predicted RNA secondary structure is close to that in the ground-truth RNA secondary structure in the training set. The setting of λ also achieves a trade-off between precision and recall of predictions through setting an appropriate λ in the potential function used by KnotFold. As demonstrated in Supplementary Fig. 6a, when set λ as 4.2, the precision and recall over valid set also achieve a balance.

Since the setting of λ is highly related to the number of base pairs, we can also modify the structural potential function (shown in Equation 1) and regard λ as a function of the number of base pairs. The high correlation between the length of the RNA sequence and the number of expected base pairs enables us to appropriately set λ prior to running KnotFold according to RNA length. More specifically, the optimal λ exhibits a highly negative linear correlation with RNA length (Pearson correlation coefficient: 0.988, shown in Supplementary Fig. 6b).

We have implemented an adaptive version of the minimum-cost flow algorithm to derive the λ value according to the length of the RNA. Specifically, according to the observation of the negative linear correlation between the optimal λ value and RNA length in Supplementary Fig. 5b, we implemented a new version of the minimum-cost flow algorithm and set λ to 4.80, 4.68, 4.60, 4.15, 3.86, 2.05, and -0.70 for RNAs with lengths falling within the ranges of 1-100, 101-200, 201-300, 301-400, 401-500, 501-1000, and longer than 1000, respectively. The results of this algorithm are very promising: its accuracy is 0.763 on PKTest dataset, higher than the original version (0.758).

As the RNAs in PKTest dataset are relatively short with length less than 500 bases, we further examined the improvement of this algorithm on long RNAs. For the 49 RNAs in the validation set with length exceeding 500 bases, the accuracy increased from 0.119 to 0.207. These results demonstrate the

potential of the adaptive λ approach to improve the accuracy of secondary structure predictions, particularly for long RNA sequences.

1.7 Investigate KnotFold's robustness using TestSetA and TestSetB

To investigate the robustness of our models, we validate them on datasets that have been widely used in previous studies. We conduct experiments following the settings employed in TORNADO [7]. Specifically, we retrained KnotFold using TrainSetA (containing 3,166 RNAs), and tested it on TestSetA (containing 592 RNAs) and TestSetB (containing 430 RNAs). We summarized the evaluation results in Supplementary Table 9. For the sake of completeness, we also listed the performance of other five approaches on these datasets, including TORNADO, ContextFold, CONTRAfold, MXfold, and MXfold2.

As a result, the retrained KnotFold achieves an F1 score of 0.728 on TestSetA and 0.551 on TestSetB, which are comparable with the performance of TORNADO (0.746 on TestSetA, 0.552 on TestSetB) and other four approaches. These findings illustrate the robustness and effectiveness of our models in accurately predicting secondary structures on widely-used datasets, even in the absence of pseudoknots.

1.8 Evaluate the performance of secondary structure prediction at lower sequence identity

Sequence identity is critical for judging whether there is substantial overlap between training set and test set. Here, we conducted a series of experiments using both CD-HIT [8] and MMseqs2 [9] to assess the KnotFold's performance at different sequence identity cut-off (0.80 and 0.40), and we list the results in Supplementary Table 10.

In the manuscript, we applied the sequence identity cut-off of 0.80 as the software tool used to construct training, validation, and test sets, CD-HIT, supports the sequence identify cut-off within the range [0.80, 1.00] only. Under this setting, we obtained 25,959 clusters from the non-redundant RNA sequences collected in bpRNA-1m and Rfam 14.5 databases, and further randomly divided these sequences into training set (23,819 sequences), validation set (1,131 sequences), and test set (1,009 sequences, denoted as PKTest).

To assess the performance of KnotFold and other approaches under the sequence identity cut-off of 0.40, we used MMseqs2, a widely-used tool in clustering protein sequences for efficient searching, instead of CD-HIT to cluster sequences and then construct data sets. We assessed the approaches using MMseqs2 as sequence clustering tool under various sequence identity cut-off, and summarized the results below:

- Sequence identity cut-off of 0.80: Under this setting, we acquired 27,799 clusters, about 7% more than that reported by CD-HIT. Next, we removed the 1,123 clusters that overlap with PKTest, and randomly divided the left-over into training set (25,676 sequences) and validation set (1,000 sequences).

We retrained KnotFold using the new training set and observed that, the acquired KnotFold model achieves an F1 score of 0.761, which is very close to the performance acquired using CD-HIT as clustering tool with the same sequence identity of 0.80.

- Sequence identity cut-off of 0.40: Under this setting, MMseqs2 produced 15,439 clusters. After removing the 793 clusters that overlap with the PKTest dataset, we randomly divided the left-over into a training set (13,646 sequences) and a validation set (1,000 sequences).
  We again retrained KnotFold using this training set and observed that the acquired KnotFold model achieved an F1 score of 0.512 on PKTest, lower than that acquired using the cut-off 0.80 by 0.249.
  We also assessed MXfold2 under this setting. The retrained MXfold2 model acquired using the same procedure achieved an F1 score of 0.475 on PKTest, also lower than that acquired using the cut-off of 0.80. We conjectured that the underlying reason might be the decrease of the training set size with the sequence identity cut-off.

1.9 Enhancing the predictions of SPOT-RNA using the minimum-cost flow algorithm

We investigated whether the minimum-cost flow algorithm could further enhance the predictions of the base pair probability generated by other approaches. Here, we built a variant of SPOT-RNA called "SPOT-RNA enhanced with minimum-cost flow", which predicts secondary structure by applying the minimum-cost flow on thebase pair probabilities reported by SPOT-RNA. Specifically, we constructed a structural potential (as shown in Equation 1) utilizing the output probabilities of SPOT-RNA. By employing these probabilities as priors and incorporating a constant reference, we apply the same minimum-cost flow algorithm to construct the secondary structure with the lowest potential.

As shown in Supplementary Table 8, the "SPOT-RNA enhanced with minimum-cost flow" variant reaches an accuracy of 0.590, showing an improvement of 0.011 compared to the performance of the original version of SPOT-RNA. These results demonstrate that the minimum-cost flow algorithm can be used to enhance SPOT-RNA to predict secondary structure.

2 Supplementary methods

2.1 Reformulating the potential function as the total cost of a flow

The RNA-specific structural potential we construct (Equation 1) can be reformulated as follows:

$$
\begin{aligned}
\mathrm{E}(S,x) &= -\sum_{\substack{i<j\\S_{i,j}=1}} \log\frac{P(bp_{i,j}|x)}{P(bp_{i,j}|\text{length})} - \sum_{\substack{i<j\\S_{i,j}=0}} \log\frac{1-P(bp_{i,j}|x)}{1-P(bp_{i,j}|\text{length})} + \lambda\sum_{i<j} S_{ij} \\
&= -\sum_{\substack{i<j\\S_{i,j}=1}} \log\frac{P(bp_{i,j}|x)}{P(bp_{i,j}|\text{length})} + \sum_{\substack{i<j\\S_{i,j}=1}} \log\frac{1-P(bp_{i,j}|x)}{1-P(bp_{i,j}|\text{length})} \\
&\quad -\sum_{\substack{i<j\\S_{i,j}=1}} \log\frac{1-P(bp_{i,j}|x)}{1-P(bp_{i,j}|\text{length})} - \sum_{\substack{i<j\\S_{i,j}=0}} \log\frac{1-P(bp_{i,j}|x)}{1-P(bp_{i,j}|\text{length})} + \lambda\sum_{\substack{i<j\\S_{i,j}=1}} S_{ij} + \lambda\sum_{\substack{i<j\\S_{i,j}=0}} S_{ij} \\
&= (-\sum_{\substack{i<j\\S_{i,j}=1}} \log\frac{P(bp_{i,j}|x)}{P(bp_{i,j}|\text{length})} + \sum_{\substack{i<j\\S_{i,j}=1}} \log\frac{1-P(bp_{i,j}|x)}{1-P(bp_{i,j}|\text{length})} + \lambda\sum_{\substack{i<j\\S_{i,j}=1}} 1) + \lambda\sum_{\substack{i<j\\S_{i,j}=0}} 0 \\
&\quad -(\sum_{\substack{i<j\\S_{i,j}=1}} \log\frac{1-P(bp_{i,j}|x)}{1-P(bp_{i,j}|\text{length})} + \sum_{\substack{i<j\\S_{i,j}=0}} \log\frac{1-P(bp_{i,j}|x)}{1-P(bp_{i,j}|\text{length})}) \\
&= \sum_{\substack{i<j\\S_{i,j}=1}} (-\log\frac{P(bp_{i,j}|x)}{P(bp_{i,j}|\text{length})} + \log\frac{1-P(bp_{i,j}|x)}{1-P(bp_{i,j}|\text{length})} + \lambda) - \sum_{i<j} \log\frac{1-P(bp_{i,j}|x)}{1-P(bp_{i,j}|\text{length})} \\
&= \sum_{\substack{i<j\\S_{i,j}=1}} H(i,j) + C
\end{aligned}
$$

Here, $H(i,j) = -[\log\frac{P(bp_{i,j}|x)}{P(bp_{i,j}|\text{length})} - \log\frac{1-P(bp_{i,j}|x)}{1-P(bp_{i,j}|\text{length})}] + \lambda$, and $C = -\sum_{i<j}\log\frac{1-P(bp_{i,j}|x)}{1-P(bp_{i,j}|\text{length})}$, which is a constant independent of structure S.

Interestingly, we observe that although the potential is calculated by adding up the contributions of all possible pairs of bases, it is essentially determined by the base pairs appearing in the secondary structure only, as the first term illustrated. This observation is consistent with conventional thermodynamic prediction methods that only consider the contribution of base pairs when calculating free energy [10–12].

By leveraging this observation, we transform the goal of finding the secondary structure with the lowest potential into calculating the minimum-cost flow in an appropriately-designed network. In particular, the network consists of a bipartite, and each part of the bipartite consists of L nodes that represent the L bases of the target RNA. We connected every node in the left part to every node in the right part with an edge, which essentially represents a possible base pair. For the edge (i,j) connecting the i-th base and the j-th base, we set its capacity as 1 and its cost as $H(i,j)$. This way, the potential exactly

describes the total cost of a flow in the constructed graph network, and the secondary structure with the lowest potential corresponds to the minimum-cost flow.

2.2 The construction of the variant KnotFold-DP

We further developed a variant of KnotFold (called KnotFold-DP), which is particularly designed for non-pseudoknot RNAs. KnotFold-DP employs the dynamic programming algorithm instead of the minimum-cost flow algorithm to determine the optimal secondary structure. For the potential function we previously constructed (Equation 1), the minimum-cost flow algorithm constructs an arbitrary secondary structure with the lowest potential, as is demonstrated above. However, when restricting RNAs to nested base pairs only, i.e. non-pseudoknot RNAs, a dynamic programming algorithm could be applied to construct the lowest potential structure.

The dynamic programming algorithm used by KnotFold-DP solves the following recursive equation: given the calculated pairwise potentials $H(i, j)$, the structure potential from the $i-th$ base to the $j-th$ base $\mathrm{E}_{i,j}$ is calculated as below:

$$\mathrm{E}_{i,j} = \min \begin{cases} \mathrm{E}_{i,j-1} \\ \min_{i \le k < j} [\mathrm{E}_{i,k-1} + \mathrm{E}_{k+1,j-1} + H(k,j)] \end{cases} \tag{1}$$

This simple DP strategy constructs a secondary structure with the lowest potential for non-pseudoknot RNAs.

2.3 The construction of the variant KnotFold-LP

We have performed an additional variant of KnotFold (called KnotFold-LP), which employs linear programming technique used in UFold [13] instead of minimum-cost flow to construct secondary structures. Using the same base pair probability as KnotFold, the linear programming algorithm constructs secondary structure from base pair probabilities with several strict constraints, and it determines the final base pairs by using an iterative grid search strategy.

As listed in Supplementary Table 8, KnotFold combinded with linear programming technique achieves an F1 score of 0.705 on PKTest, lower than the performance of the combination with the minimum-cost flow technique by 0.053. This comparison clearly illustrates the advantages of the minimum-cost flow algorithm over the linear programming approach used by UFold.

3 Supplementary figures and tables

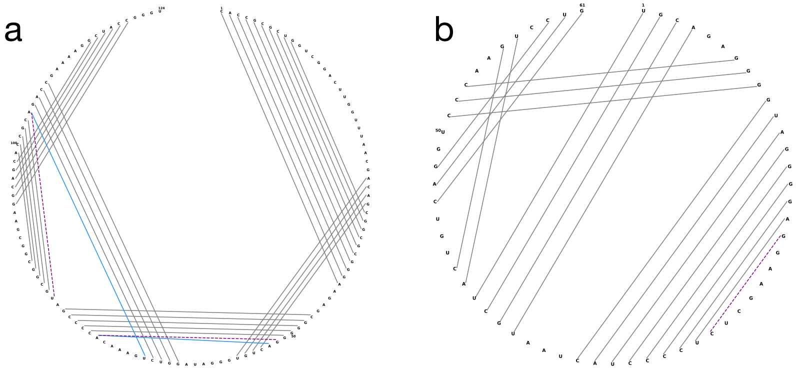

Supplementary Fig. 1 The predicted secondary structures by KnotFold on two RNAs with K- and L-type pseudoknots. a, The predicted structure for the RNA `URS0000D69A17_12908_1-124` with the K-type pseudoknot. **b**, The predicted structure for the RNA `URS0000D67D31_12908_1-61` with L-type pseudoknots. In both cases, KnotFold can successfully identify these pseudoknots. Here, magenta dash lines represent the missing base pairs and blue lines represent the false-positive base pairs

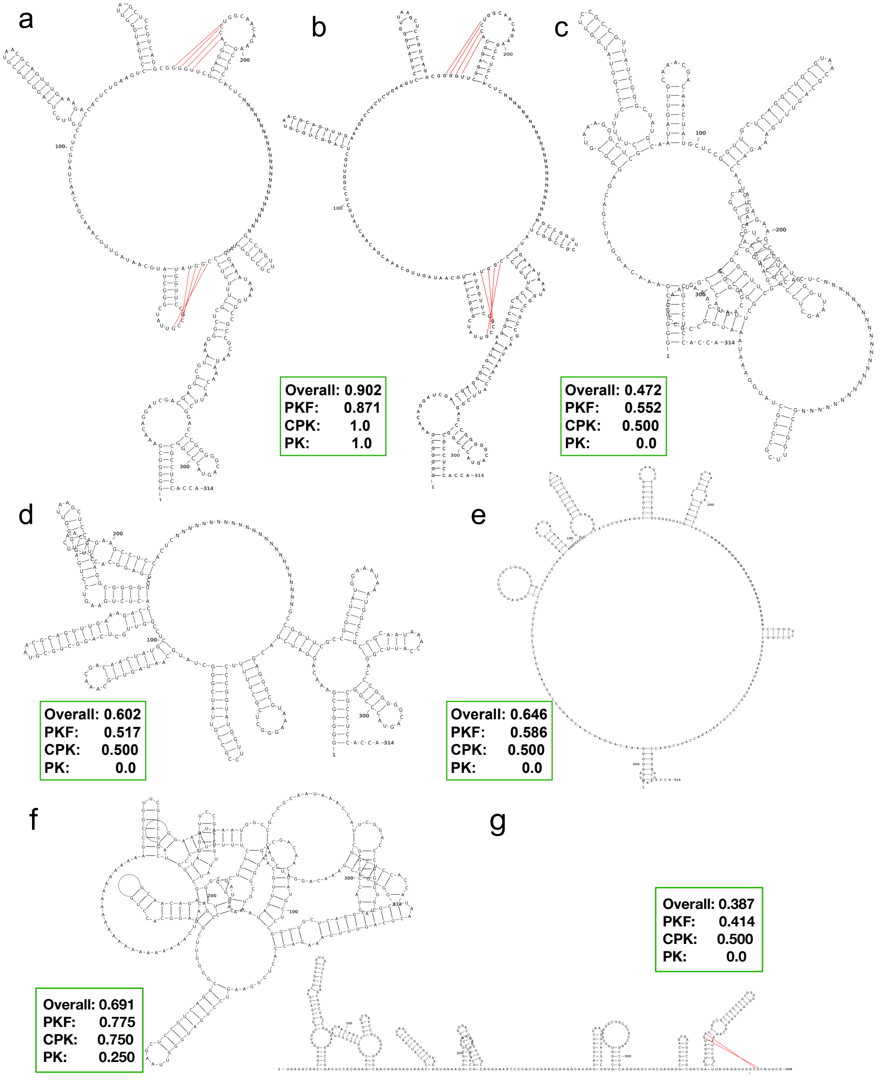

Supplementary Fig. 2 Predicted secondary structures by KnotFold, MXfold2, ProbKnot, SPOT-RNA, UFold and IPknot (target RNA: `TRW-314253_1-314`**).** **a**, The ground-truth secondary structure, in which the pseudoknotted base pairs are shown in red. **b**, The structure predicted by KnotFold with an overall F1 of 0.902. **c**, The structure predicted by MXfold2 with an overall F1 of 0.472. **d**, The structure predicted by ProbKnot with an overall F1 of 0.602. **e**, The structure predicted by SPOT-RNA with an overall F1 of 0.646. **f**, The structure predicted by UFold with an overall F1 of 0.691. **g**, The structure predicted by IPknot with an overall F1 of 0.387.

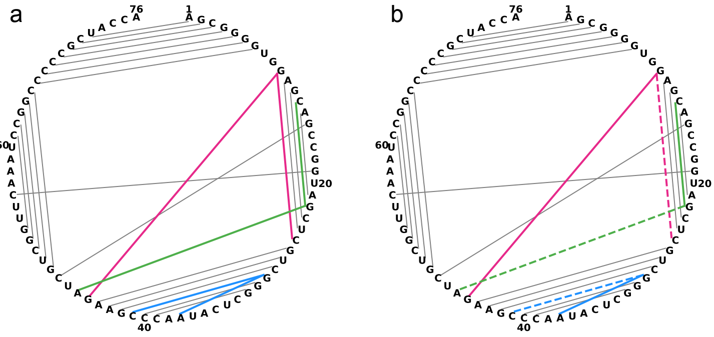

Supplementary Fig. 3 Predicted RNA secondary structures including base triples using the enhanced KnotFold (RNA: PDB entry 5L40). a, The predicted structure for the RNA with PDB entry 5L40 using the enhanced KnotFold, in which the three base triples 25C-10G-45G (in magenta), 13C-22G-46A (in green), and 37A-29G-41C (in blue) are successfully identified. **b**, The predicted secondary structure by the original KnotFold without enhancement. The missing base pairs 25C-10G, 22G-46A and 29G-41C (dashed lines) lead to the failure in identifying the base triples

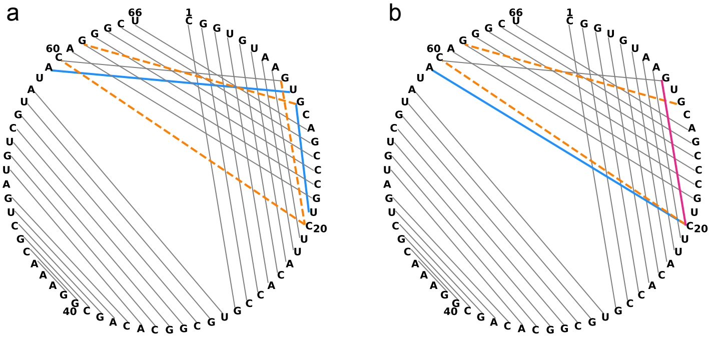

Supplementary Fig. 4 Predicted secondary structures including base triples using the enhanced KnotFold (RNA: PDB entry 7LYJ). a, The predicted secondary structure by the original KnotFold without enhancement. Orange dash lines represent the missing base pairs or base triples and blue lines represent the false-positive base pairs. **b**, The predicted secondary structure using the enhanced KnotFold. The base triple 20C-9G-60C was successfully identified by KnotFold (in magenta)

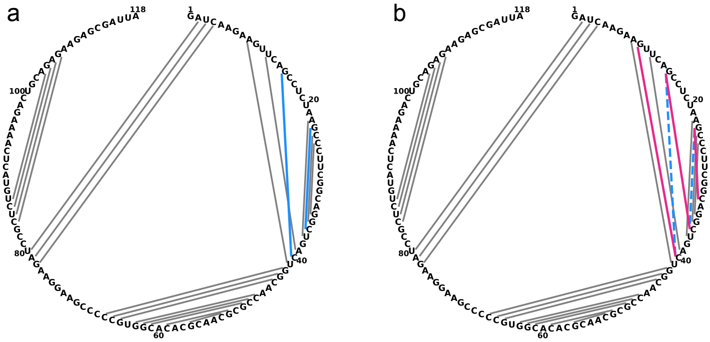

Supplementary Fig. 5 Removing tentatively-selected base pairs for improving prediction (target RNA: `BA000035.2_1474355-1474472`). **a**, An intermediate secondary structure reported by KnotFold for `BA000035.2_1474355-1474472`. The structure has a potential of -378.829, and it contains a total of 22 base pairs, two of which will be removed in the next iteration step (shown as blue lines). **b**, The secondary structure reported by KnotFold after executing an iteration of improvement, in which three base pairs are newly added (in magenta) and two base pairs are removed (in blue, dashed lines). The structure has a potential of -379.831, lower than the previous intermediate structure before this iteration step

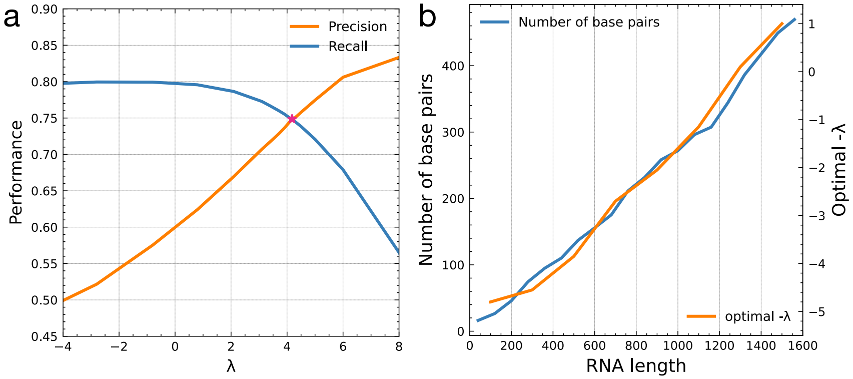

Supplementary Fig. 6 Optimal setting of parameter λ in the structural potential function. a, Average accuracy (precision and recall) over the validation set versus the value of λ (1,131 RNAs). When λ is set as 4.2, which makes average number of base pairs expected to appear in the predicted RNA secondary structure close to that in the ground truth RNA secondary structure in the training set, it also achieves a balance of precision and recall on the validation set. **b**, Optimal setting of λ over RNA length. Pearson's correlation coefficient between the negative of optimal λ (the orange curve) and RNA length (the blue curve) reaches 0.988

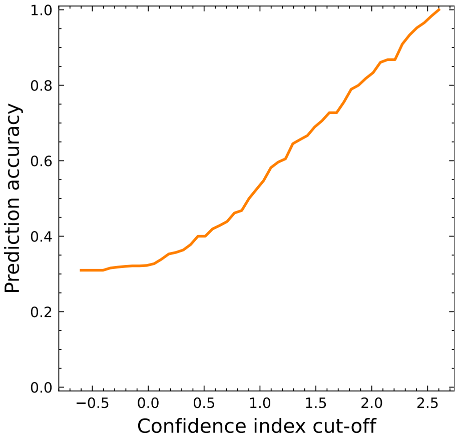

Supplementary Fig. 7 The prediction accuracy at different confidence index cut-off (confidence level: 95%). The prediction accuracy together with the confidence index are calculated using the 1131 RNAs in the validation set. For example, the point $(1.35, 0.60)$ in the curve means that, if an RNA has its confidence index estimated to be over 1.35, we can deduce that, at the confidence level of 95%, the prediction accuracy for this RNA exceeds 0.60

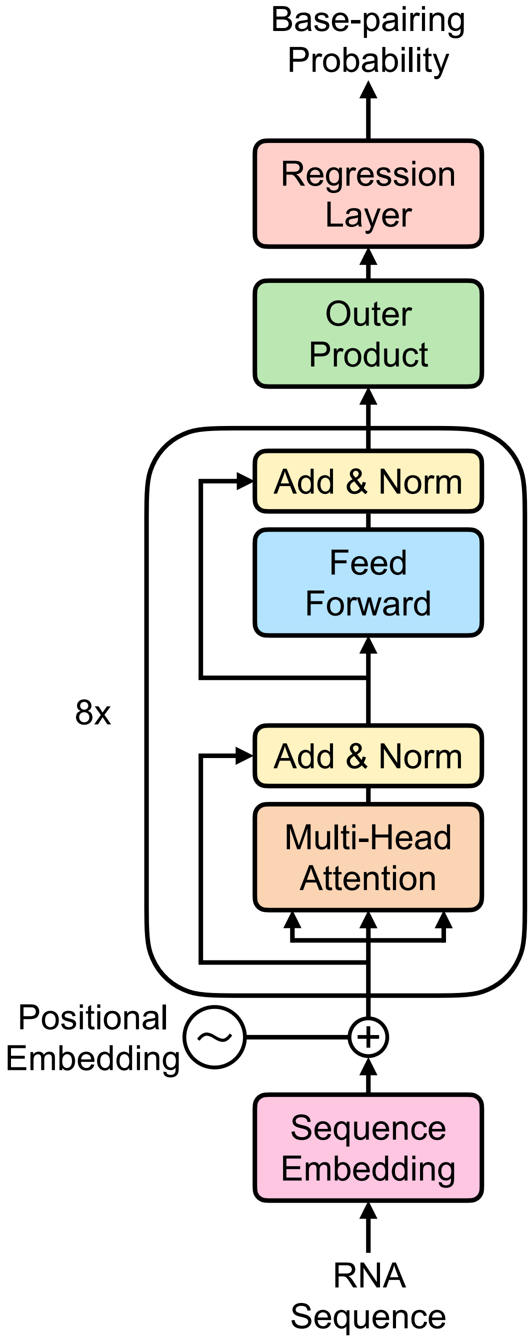

Supplementary Fig. 8 The network architecture used by KnotFold for predicting base pairing probability. The network first embeds the input RNA sequence, feeds the sequence embedding and the position embedding into transformer encoder blocks, and finally utilizes an outer product operator followed by a regression layer to obtain base pairing probabilities

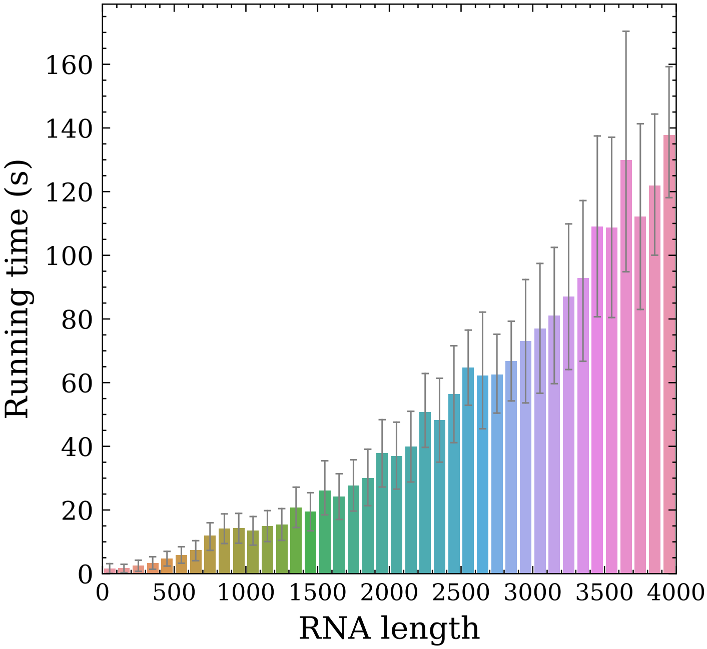

Supplementary Fig. 9 The running time of the minimum-cost flow algorithm for RNAs with different length in the validation set. The running time is evaluated on 1,131 RNAs in the validation set. The minimum-cost flow algorithm constructs the secondary structures within 20 seconds for RNAs no longer than 1,000 bases, and within 50 seconds for RNAs no longer than 2,000 bases

Supplementary Table 1 The accuracy of secondary structure prediction approaches for different complexity of pseudoknot structures on PKTest

Method	Overall	H-type	K-type	L, M-type
KnotFold	0.758	0.713	0.782	0.783
UFold	0.578	0.596	0.564	0.476
UFold (retrained)	0.602	0.601	0.614	0.598
SPOT-RNA	0.579	0.582	0.594	0.541
MXfold2	0.487	0.473	0.542	0.495
MXfold2 (retrained)	0.498	0.476	0.539	0.509
IPknot	0.483	0.477	0.523	0.451
RNAstructure	0.449	0.436	0.501	0.447
ProbKnot	0.451	0.437	0.505	0.457
HotKnots	0.489	0.485	0.503	0.480
pKiss	0.421	0.442	0.367	0.344
Knotty	0.339	0.379	0.258	0.211

Supplementary Table 2 The predicted accuracy of secondary structure prediction approaches for pseudoknot-free base pairs, crossing-pseudoknot base pairs, and pseudoknotted base pairs on PKTest (1,009 RNAs)

Method	Accuracy for PKF base pairs	Accuracy for CPK base pairs	Accuracy for PK base pairs
KnotFold	0.697	0.783	0.734
UFold	0.592	0.628	0.172
UFold (retrained)	0.581	0.634	0.304
SPOT-RNA	0.536	0.566	0.242
MXfold2	0.493	0.521	0.130
MXfold2 (retrained)	0.495	0.509	0.138
IPknot	0.448	0.462	0.188
RNAstructure	0.473	0.432	0.144
ProbKnot	0.484	0.450	0.172
HotKnots	0.467	0.512	0.258
pKiss	0.390	0.454	0.260
Knotty	0.300	0.382	0.236

Supplementary Table 3 The predicted accuracy of the secondary structure prediction approaches evaluated on TS0 (1,305 RNAs)

Method	F1	Precision	Recall
KnotFold	0.666	0.664	0.689
UFold	0.630	0.612	0.673
SPOT-RNA	0.596	0.579	0.651
MXfold2	0.553	0.522	0.621
IPknot	0.525	0.514	0.569
RNAstructure	0.501	0.465	0.576
ProbKnot	0.491	0.446	0.581
HotKnots	0.509	0.471	0.586
pKiss	0.440	0.400	0.515
Knotty	0.476	0.432	0.560

Supplementary Table 4 The predicted accuracy of the secondary structure prediction approaches evaluated on RfamNew (472 RNAs)

Method	F1	Precision	Recall
KnotFold	0.667	0.674	0.684
UFold	0.642	0.670	0.636
SPOT-RNA	0.649	0.681	0.657
MXfold2	0.674	0.681	0.691
IPknot	0.681	0.711	0.681
RNAstructure	0.667	0.664	0.692
ProbKnot	0.661	0.657	0.691
HotKnots	0.677	0.678	0.700
pKiss	0.489	0.469	0.523
Knotty	0.524	0.501	0.562

Supplementary Table 5 The prediction accuracy of the five models within the ensemble of identical network architectures on the validation set (1,131 RNAs), PKTest (1,009 RNAs), and RfamNew (472 RNAs)

Model	F1 on the validation set	F1 on PKTest	F1 on RfamNew
Model 1	0.715	0.734	0.643
Model 2	0.711	0.730	0.649
Model 3	0.712	0.733	0.642
Model 4	0.717	0.747	0.650
Model 5	0.712	0.727	0.656
Ensemble	0.724	0.758	0.667

Supplementary Table 6 The model architecture and the prediction accuracy of the five models within the ensemble of different network architectures on the validation set, PKTest, and RfamNew

Model	#Transformer layers	#Dimension	#Paramater	F1 on the validation set	F1 on PKTest	F1 on RfamNew
Model 1	4	512	13.4M	0.721	0.723	0.646
Model 2	6	374	10.5M	0.714	0.743	0.655
Model 3	8	256	6.5M	0.717	0.747	0.650
Model 4	10	256	8.1M	0.720	0.738	0.633
Model 5	12	256	9.6M	0.709	0.726	0.638
Ensemble	-	-	-	0.732	0.765	0.671

Supplementary Table 7 Accuracy of the predicted base pair probabilities generated by KnotFold's prior model, SPOT-RNA, and UFold on PKTest

Method	Overall accuracy	Accuracy for PKF base pairs	Accuracy for CPK base pairs	Accuracy for PK base pairs	Accuracy for long range base pairs
KnotFold	0.729	0.697	0.771	0.687	0.535
SPOT-RNA	0.568	0.552	0.596	0.226	0.285
UFold	0.561	0.584	0.629	0.158	0.264
UFold (retrained)	0.576	0.592	0.583	0.292	0.185

Supplementary Table 8 Accuracy of the predicted secondary structure by KnotFold, SPOT-RNA and their variants on PKTest. Here, KnotFold-DP uses dynamic programming instead of the minimum-cost flow technique. KnotFold-LP uses the linear programming proposed by UFold instead of the minimum-cost flow algorithm. 'SPOT-RNA enhanced with minimum-cost flow' predicts secondary structure by applying the minimum-cost flow on the base pair probabilities reported by SPOT-RNA

Method	Overall accuracy	Accuracy for PKF base pairs	Accuracy for CPK base pairs	Accuracy for PK base pairs
KnotFold	0.758	0.697	0.783	0.734
KnotFold-DP	0.708	0.716	0.698	0.176
KnotFold-LP	0.705	0.707	0.744	0.621
SPOT-RNA enhanced with minimum-cost flow	0.590	0.574	0.620	0.295
SPOT-RNA	0.579	0.536	0.566	0.242

Supplementary Table 9 The predicted accuracy of KnotFold, TORNADO, ContextFold, CONTRAfold, MXfold, and MXfold2. These approaches are trained on TrainSetA, and tested on TestSetA and TestSetB

Method	F1 score on TestSetA	F1 score on TestSetB
KnotFold	0.728	0.551
TORNADO	0.746	0.552
ContextFold	0.759	0.502
CONTRAfold	0.719	0.573
MXfold2	0.761	0.601
MXfold	0.739	0.581

Supplementary Table 10 The predicted accuracy of KnotFold at different sequence identity thresholds using CD-HIT and MMseqs2

Clustering method	Sequence identity cut-off	Non-redundant clusters	Training data size	Validation data size	F1 on PKTest	F1 on RfamNew
CD-HIT	0.80	25,959	23,819	1,131	0.758	0.667
MMseqs2	0.80	27,799	25,676	1,000	0.761	0.681
MMseqs2	0.40	15,439	13,646	1,000	0.512	0.522

Supplementary Table 11 The number of pseudoknot base pairs in PKTest and RfamNew

Dataset	#RNAs	#Pseudoknotted RNAs	#Pseudoknot base pairs	Average sequence length	Maximum sequence length
PKTest	1,009	1,009	1,194	186	492
RfamNew	472	51	301	115	464

Supplementary Table 12 The number of base triples in TR1, VL1, TS1 and TS2

Dataset	#RNAs	#RNAs containing base triples	Multiplets base pairs
TR1	120	104	1,194
VL1	30	27	301
TS1	67	53	406
TS2	39	29	230

Supplementary Table 13 Predicted accuracy of KnotFold and SPOT-RNA on TS1 and TS2

Method	F1 on TS1	Accuracy for base triples on TS1	F1 on TS2	Accuracy for base triples on TS2
KnotFold	0.739	0.239	0.841	0.347
SPOT-RNA	0.714	0.192	0.810	0.211

Supplementary References

[1] Legendre, A., Angel, E., Tahi, F.: Bi-objective Integer Programming for RNA Secondary Structure Prediction with Pseudoknots. BMC Bioinformatics **19**(1), 1–15 (2018)

[2] Abu Almakarem, A.S., Petrov, A.I., Stombaugh, J., Zirbel, C.L., Leontis, N.B.: Comprehensive Survey and Geometric Classification of Base Triples in RNA Structures. Nucleic Acids Research **40**(4), 1407–1423 (2012)

[3] Wang, S., Ke, H., Zhang, H., Ma, Y., Ao, L., Zou, L., Yang, Q., Zhu, H., Nie, J., Wu, C., *et al.*: LncRNA MIR100HG Promotes Cell Proliferation in Triple-negative Breast Cancer through Triplex Formation with P27 Loci. Cell Death & Disease **9**(8), 1–11 (2018)

[4] Devi, G., Zhou, Y., Zhong, Z., Toh, D.-F.K., Chen, G.: RNA Triplexes: from Structural Principles to Biological and Biotech Applications. Wiley Interdisciplinary Reviews: RNA **6**(1), 111–128 (2015)

[5] Dietterich, T.G.: Ensemble Methods in Machine Learning. In: International Workshop on Multiple Classifier Systems, pp. 1–15 (2000). Springer

[6] Doshi, K.J., Cannone, J.J., Cobaugh, C.W., Gutell, R.R.: Evaluation of the Suitability of Free-energy Minimization using Nearest-Neighbor Energy Parameters for RNA Secondary Structure Prediction. BMC Bioinformatics **5**(1), 1–22 (2004)

[7] Rivas, E., Lang, R., Eddy, S.R.: A Range of Complex Probabilistic Models for RNA Secondary Structure Prediction that Includes the Nearest-neighbor Model and More. RNA **18**(2), 193–212 (2012)

[8] Fu, L., Niu, B., Zhu, Z., Wu, S., Li, W.: CD-HIT: Accelerated for Clustering the Next-generation Sequencing Data. Bioinformatics **28**(23), 3150–3152 (2012)

[9] Steinegger, M., Söding, J.: MMseqs2 Enables Sensitive Protein Sequence Searching for the Analysis of Massive Data Sets. Nature Biotechnology **35**(11), 1026–1028 (2017)

[10] Turner, D.H., Mathews, D.H.: NNDB: the Nearest Neighbor Parameter Database for Predicting Stability of Nucleic Acid Secondary Structure. Nucleic Acids Research **38**(suppl_1), 280–282 (2010)

[11] Zuker, M., Stiegler, P.: Optimal Computer Folding of Large RNA Sequences Using Thermodynamics and Auxiliary Information. Nucleic Acids Research **9**(1), 133–148 (1981)

[12] Mathews, D.H., Andre, T.C., Kim, J., Turner, D.H., Zuker, M.: An Updated Recursive Algorithm for RNA Secondary Structure Prediction with Improved Thermodynamic Parameters. ACS Publications (1998)

[13] Fu, L., Cao, Y., Wu, J., Peng, Q., Nie, Q., Xie, X.: UFold: Fast and Accurate RNA Secondary Structure Prediction with Deep Learning. Nucleic Acids Research **50**(3), 14–14 (2022)
