# Supplementary material for: Accurate prediction of RNA secondary structure including pseudoknots through solving minimum-cost flow with learned potentials
Source: Commun Biol. 2024 Mar 9;7:297. doi: 10.1038/s42003-024-05952-w (PMC10924946; doi:10.1038/s42003-024-05952-w)
Supplement: Supplementary file 2 — Description of Supplementary Materials [file 42003_2024_5952_MOESM2_ESM.docx]

**Description of Additional Supplementary Files**

**File name:** Supplementary Data 1

**Description:** Training and test datasets.
