# Supplementary material for: Accurate prediction of RNA secondary structure including pseudoknots through solving minimum-cost flow with learned potentials
Source: Commun Biol. 2024 Mar 9;7:297. doi: 10.1038/s42003-024-05952-w (PMC10924946; doi:10.1038/s42003-024-05952-w)
Supplement: Supplementary file 4 — Reporting Summary [file 42003_2024_5952_MOESM4_ESM.pdf]

Corresponding author(s): Dongbo Bu

Last updated by author(s): Feb 1, 2024

Reporting Summary

Nature Portfolio wishes to improve the reproducibility of the work that we publish. This form provides structure for consistency and transparency in reporting. For further information on Nature Portfolio policies, see our Editorial Policies and the Editorial Policy Checklist.

Statistics

For all statistical analyses, confirm that the following items are present in the figure legend, table legend, main text, or Methods section.

n/a	Confirmed	
☐	☒	The exact sample size (n) for each experimental group/condition, given as a discrete number and unit of measurement
☒	☐	A statement on whether measurements were taken from distinct samples or whether the same sample was measured repeatedly
☒	☐	The statistical test(s) used AND whether they are one- or two-sided<br>*Only common tests should be described solely by name; describe more complex techniques in the Methods section.*
☒	☐	A description of all covariates tested
☒	☐	A description of any assumptions or corrections, such as tests of normality and adjustment for multiple comparisons
☐	☒	A full description of the statistical parameters including central tendency (e.g. means) or other basic estimates (e.g. regression coefficient) AND variation (e.g. standard deviation) or associated estimates of uncertainty (e.g. confidence intervals)
☒	☐	For null hypothesis testing, the test statistic (e.g. F, t, r) with confidence intervals, effect sizes, degrees of freedom and P value noted<br>*Give P values as exact values whenever suitable.*
☒	☐	For Bayesian analysis, information on the choice of priors and Markov chain Monte Carlo settings
☒	☐	For hierarchical and complex designs, identification of the appropriate level for tests and full reporting of outcomes
☐	☒	Estimates of effect sizes (e.g. Cohen's d, Pearson's r), indicating how they were calculated

Our web collection on statistics for biologists contains articles on many of the points above.

Software and code

Policy information about availability of computer code

Data collection	The datasets used in our experiments were downloaded from:<br>bpRNA: http://bprna.cgrb.oregonstate.edu/<br>Rfam: https://rfam.xfam.org/<br>To build our datasets, we removed redundant sequences collected from bpRNA-1m 1.0 and Rfam 14.5 using CD-HIT-EST with a cutoff threshold of 80% sequence-identity, and then randomly split the remaining data into the training, validation, and test sets.<br>We constructed an RNA family test set RfamNew from the 168 families that are newly added to Rfam 14.9 after the release of version 14.5.
Data analysis	Predictions of RNA secondary structures were performed by the following software packages:<br>RNAstructure version 6.4,<br>Knotty (committed on Mar 29, 2018),<br>pKiss (built on June 25 2015),<br>IPknot (committed on Mar 1, 2023),<br>SPOT-RNA (committed on Jan 24, 2022),<br>MXfold2 (committed on Jun 22, 2021),<br>UFold (committed on Oct 14, 2022),<br>and our software KnotFold available at https://github.com/gongtiansu/KnotFold

For manuscripts utilizing custom algorithms or software that are central to the research but not yet described in published literature, software must be made available to editors and reviewers. We strongly encourage code deposition in a community repository (e.g. GitHub). See the Nature Portfolio guidelines for submitting code & software for further information.

Data

Policy information about availability of data

All manuscripts must include a data availability statement. This statement should provide the following information, where applicable:

- Accession codes, unique identifiers, or web links for publicly available datasets
- A description of any restrictions on data availability
- For clinical datasets or third party data, please ensure that the statement adheres to our policy

The data used by KnotFold for training, testing and validation is publicly available at github (https://github.com/gongtiansu/KnotFold).

Human research participants

Policy information about studies involving human research participants and Sex and Gender in Research.

Reporting on sex and gender

Use the terms sex (biological attribute) and gender (shaped by social and cultural circumstances) carefully in order to avoid confusing both terms. Indicate if findings apply to only one sex or gender; describe whether sex and gender were considered in study design whether sex and/or gender was determined based on self-reporting or assigned and methods used. Provide in the source data disaggregated sex and gender data where this information has been collected, and consent has been obtained for sharing of individual-level data; provide overall numbers in this Reporting Summary. Please state if this information has not been collected. Report sex- and gender-based analyses where performed, justify reasons for lack of sex- and gender-based analysis.

Population characteristics

Describe the covariate-relevant population characteristics of the human research participants (e.g. age, genotypic information, past and current diagnosis and treatment categories). If you filled out the behavioural & social sciences study design questions and have nothing to add here, write "See above."

Recruitment

Describe how participants were recruited. Outline any potential self-selection bias or other biases that may be present and how these are likely to impact results.

Ethics oversight

Identify the organization(s) that approved the study protocol.

Note that full information on the approval of the study protocol must also be provided in the manuscript.

Field-specific reporting

Please select the one below that is the best fit for your research. If you are not sure, read the appropriate sections before making your selection.

☒ Life sciences ☐ Behavioural & social sciences ☐ Ecological, evolutionary & environmental sciences

For a reference copy of the document with all sections, see nature.com/documents/nr-reporting-summary-flat.pdf

Life sciences study design

All studies must disclose on these points even when the disclosure is negative.

Sample size

The sample size of training, testing and validation set was determined by the number of sequences that were identified as non-redundant by CD-HIT-EST among the 102,318 sequences from bpRNA-1m and the 4108 families of seed alignments that were registered after the Rfam version 14.9.

Data exclusions

All redundant sequences homologous to each other were excluded by using CD-HIT-EST version 4.8.1 with 80% sequence identity cutoff.

Replication

All the training weights/ parameters of the models were saved during experiments. These trained parameters are deployed at github (https://github.com/gongtiansu/KnotFold) for results replication. All these model parameters are available as standalone software to run on a local computer for results replication.

Randomization

All the non-redundant sequences were randomly split into training, testing and validation data sets.

Blinding

The test sets and the validation set used for performance measures were completely independent of datasets used for model training. CD-HIT-EST tool was used to separate test sets from training data.

Reporting for specific materials, systems and methods

We require information from authors about some types of materials, experimental systems and methods used in many studies. Here, indicate whether each material, system or method listed is relevant to your study. If you are not sure if a list item applies to your research, read the appropriate section before selecting a response.

Materials & experimental systems

n/a	Involved in the study
☒	☐ Antibodies
☒	☐ Eukaryotic cell lines
☒	☐ Palaeontology and archaeology
☒	☐ Animals and other organisms
☒	☐ Clinical data
☒	☐ Dual use research of concern

Methods

n/a	Involved in the study
☒	☐ ChIP-seq
☒	☐ Flow cytometry
☒	☐ MRI-based neuroimaging
